# Clinical Intervention Strategies and Family Dynamics in Adolescent Eating Disorders: A Scoping Review for Enhancing Early Detection and Outcomes

**DOI:** 10.3390/jcm13144084

**Published:** 2024-07-12

**Authors:** Evgenia Gkintoni, Elias Kourkoutas, Stephanos P. Vassilopoulos, Maria Mousi

**Affiliations:** 1 Department of Education and Social Work, University of Patras, 26504 Patras, Greece; stephanosv@upatras.gr; 2 Department of Primary Education, Research Center for the Humanities, Social and Education Sciences, University of Crete, 74150 Rethymno, Greece; eliaskourk@uoc.gr; 3 Department of Psychology, University of Crete, 74150 Rethymno, Greece; mariamousipsy@gmail.com

**Keywords:** eating disorders, adolescents, family, psychotherapeutic interventions, pharmacological therapy

## Abstract

**Background**: This systematic review investigated the impact of familial factors on individuals aged 10–17 who have clinical signs or symptoms of eating disorders. Simultaneously, it scrutinized the involvement of the family in therapy, as well as other forms of intervention. **Methods**: The PsycINFO, PubMed, and Scopus databases were used to search for research material comprehensively. After applying specific criteria, 46 articles were deemed suitable and included in the systematic review. The study comprised a cohort of 4794 adolescents who received a diagnosis of either Anorexia Nervosa (AN), Bulimia Nervosa (BN), or Binge-Eating Disorder (BED). In addition, controls were utilized for 1187 adolescents, 1563 parents, 1809 siblings, and 11 other relatives. **Results**: The connection between family factors and eating disorders is primarily determined by the families’ level of functioning, satisfaction with the family dynamic, parents’ attitudes toward their children, and the role of food within the family system. Family Therapy was the most used psychotherapeutic approach in the treatment of AN. The incidence of reports in BN closely paralleled that of Cognitive–Behavioral Therapy (CBT) models. Articles about (Enhanced) CBT were exclusively associated with BED. **Conclusions**: Family-based approaches are crucial in comprehending, preventing, and addressing eating disorders in adolescents. Incorporating the study of family dynamics and actively engaging families in the treatment process can significantly enhance recovery rates and decrease the occurrence of relapses.

## 1. Introduction

Eating disorders usually develop in adolescents aged from 14 to 19 [1]. According to research [2], these are severe mental disorders that impact many individuals globally, regardless of their race, age, ethnicity, or gender. These disorders have significant effects on individual mental functioning and interpersonal relationships, as well as on families and society. Research in comorbidity and mortality is limited due to the higher cost involved. As a result, there has been a shift in research focus toward understanding their pathophysiological and neurobiological aspects [3]. Researchers [4,5,6] state that only a small number of interventions have been identified as being definitively effective, particularly in the case of adolescents. Most of the research conducted in this field and with this group of individuals primarily emphasizes the role of the family in treatment, given its paramount significance during the developmental phase of those affected [6].

Adolescent eating disorders are multifaceted disorders that are affected by multiple factors, such as cognitive, socio-emotional, and interpersonal elements [7]. The cognitive–interpersonal maintenance model of Anorexia Nervosa (AN) posits that these factors interact to both initiate and sustain eating disorders. An essential characteristic of this model is the diminished ability to switch between tasks and the limited ability to process information holistically, which are linked to obsessive–compulsive traits and may be hereditary predispositions to AN [7]. In addition, individuals with AN may experience challenges in socio-emotional processing, such as showing a tendency to focus on critical facial expressions and having difficulties in controlling their emotions. Family dynamics and relationships exert a substantial influence on the development of eating disorders in adolescents. Excessive expression of emotion, which includes criticism, hostility, and overprotection, can unintentionally sustain the problem [8]. Empirical evidence has confirmed that the functioning of the family plays a significant role in the emergence and persistence of eating disorders among adolescents [6,7,8].

The significance of family relationships, specifically family functioning, has been recognized and investigated in eating disorders. When comprehending adolescents’ distress and devising successful interventions, it is crucial to consider familial circumstances [8]. Obese adolescents have a higher likelihood of developing eating disorders. The risk factors for eating disorders in this group consist of body dissatisfaction, low self-esteem, depression, and involvement in dieting practices [9]. Engaging in unsupervised dieting to control weight can worsen the likelihood of developing an eating disorder, whereas following a structured and supervised weight-management program can decrease the risk for most adolescents [9].

Nevertheless, teenagers who are obese may exhibit undisclosed eating disorders or develop eating disorders while undergoing treatment or after receiving treatment. Efforts to prevent eating disorders should focus on early adolescence, specifically on 14-year-old girls, as research suggests that this is the age group where risk factors are most indicative of the future development of eating disorders [10]. Research conducted by [10] has shown that feeling dissatisfied with one’s body is a reliable indicator of the early onset of eating disorders. It is recommended to employ screening and monitoring protocols to identify individuals who are at risk of developing eating disorders, particularly adolescents who are obese [9]. Promoting positive family relationships, psychological well-being, and regular meals is crucial, while discouraging excessive emphasis on weight and unhealthy weight-control behaviors [7,8,9,10].

This article aims to examine the influence of family dynamics on adolescents between the ages of 10 and 17 who exhibit clinical indications or symptoms of eating disorders. Additionally, it explores the significance of family engagement in therapy and various types of intervention. The objective of the paper is to improve the comprehension and results of early identification and therapy by examining the pertinent literature and synthesizing findings on familial impact and the efficacy of different psychotherapeutic and pharmacological interventions for eating disorders such as AN, BN, and Binge-Eating Disorder (BED).

## 2. Literature Review

### 2.1. Anorexia Nervosa [AN]

#### 2.1.1. Psychotherapeutic Interventions

The Maudsley Approach, also known as Family-Based Therapy (FBT), is the most effective treatment method for adolescents with AN [11]. Modifying disruptive and maladaptive family environments makes it feasible to attain swift progress with enduring results, with a potential success rate of up to 90%. The intervention lasts 9–12 months and is implemented through sessions divided into three phases [12]. More precisely, during the initial phase, the sessions occur every week; they are held twice a month in the second phase; and, finally, they occur once a month in the third phase. Researchers conducted a meta-analysis and concluded that FBT is more effective than individual therapies within 6–12 months after the intervention [13]. Nevertheless, there was no statistically significant correlation immediately after the intervention concluded. This intervention aims to reduce the patient’s weight to a normal range, regulate his/her eating habits, and help him/her reintegrate into typical adolescent growth and maturation [14]. Parents actively participate, to different extents, in all stages of treatment. During the initial stage, they assume complete accountability for the patient’s dietary regimen, encompassing the specific food choices, meal timings, and feeding techniques. During the second phase, as a consistent rise in weight is observed and a heightened inclination for parental participation is noticed, the responsibility is gradually transferred to the adolescent.

Nevertheless, oversight and intervention remain necessary in the event of insufficient nourishment. During the third phase, the therapist assesses the child’s development and then informs the parents about the typical challenges of adolescence. The parents, in turn, contribute to adapting to this new information, ensuring that regression is not used as a coping mechanism.

Researchers [15] state that two sessions involve the therapist, the patient, and the patient’s family before commencing the initial treatment phase. Specifically, during the initial consultation, the specialist engages in a brief conversation with the teenager, assesses his/her weight, and subsequently involves the entire family. The specialist gathers information from each family member regarding the impact of the disorder on the family system. To achieve maximum engagement of the adolescent and alleviate the symptoms he/she is experiencing, the specialist expresses a non-judgmental acceptance, while also emphasizing to the parents the severity of the disorder to motivate them to carry out the assigned task of refeeding the patient. According to the researchers, the second session, known as “the family meal”, commences similarly to the first.

Nevertheless, during this gathering, parents are requested to choose a meal that they deem suitable for fulfilling the nutritional requirements of an underweight individual. Upon the family’s participation in the session, all individuals consume food, while the specialist evaluates their eating conduct. When the adolescent ceases consuming food, parents learn to motivate him/her to finish even a modest quantity to comprehend that they can provide more nourishment. Ultimately, the specialist instructs the parents on the nutritional therapy for their child, while also involving the siblings in the therapeutic process. The specialist clarifies that the siblings are not responsible for the parents’ duties and emphasizes their supportive role toward the teenager [16].

Enhanced CBT is another intervention method that can be moderately effective in treating adolescents with AN. Based on a study conducted by researchers [17], adolescents aged 13–19 who suffer from this condition experienced an improvement in their Body Mass Index (BMI) both after the treatment and twenty weeks after that. Despite a decrease in weight during the sixty-week reassessment, this intervention has been demonstrated to help reduce the likelihood of relapse inside and outside the hospital setting [18]. Unlike FBT, the focus is primarily on the adolescent, as it is assumed that the symptoms of the eating disorder belong to him/her [18]. Parents are included in the therapeutic intervention, but their participation is limited.

The patient is responsible for both regaining weight and managing the use of laxatives. Patients are gradually taught to handle dietary restrictions and other control behaviors and address concerns about body shape and weight. The frequency and length of the treatment vary based on the patient’s weight. Patients with lower weights undergo 40 sessions throughout a period from 9 to 12 months, while patients with higher weights undergo 20 sessions over six months [19]. The efficacy of classical CBT, which seeks to modify knowledge and behaviors associated with weight and body shape, has been demonstrated [20,21]. Two distinctions have been identified regarding the improved model: (1) The treatment lasts for three months and consists of 12–14 sessions [21]. (2) Unlike Enhanced CBT, which primarily targets adolescents, there is no adjustment made for both eating disorders and age [22].

Researchers [23] found that combining dialectical behavior therapy and FBT produces positive outcomes. During their study on adolescent girls between the ages of 13 and 18 diagnosed with AN and Atypical AN, the utilization of both methods above led to an improvement in adaptive capabilities and the attainment of the anticipated body weight. Nevertheless, as per the assessment conducted by researchers [24], Dialectical Behavior Therapy demonstrates efficacy as a standalone treatment for adolescents suffering from eating disorders. While the limited number of studies prevents generalization of the research results, potential benefits have been observed, including enhancements in BMI and reductions in the frequency of vomiting and binge eating. This approach integrates individual therapy, group skills, telephone guidance, and counseling, as one study [25] outlined. The therapist’s objective is to minimize (a) self-harming behaviors, both suicidal and non-suicidal, which are frequently observed in adolescents with AN, (b) behaviors that hinder the progress of treatment, and (c) behaviors that have an impact on the individual’s overall quality of life [26]. Following the intervention, the patient demonstrates enhanced emotional regulation [27,28]. The ability to avoid emotions and the challenge of recognizing them is highly advantageous [29].

#### 2.1.2. Pharmacological Therapy

Pharmacotherapy is an alternative treatment for AN in teenagers. Nevertheless, it is consistently utilized in conjunction with psychotherapeutic techniques, nutritional counseling, and the management of physical complications [30]. The efficacy of this intervention is subject to debate. No drug has been approved for administration by the Food and Drug Administration in the USA. According to research [31], the use of psychotropic drugs in teenagers, specifically antidepressants like paroxetine, sertraline, fluoxetine, and citalopram, as well as antipsychotics like haloperidol, olanzapine, and risperidone, can lead to an increase in appetite and weight [32]. However, these drugs primarily help in treating other mental health conditions that occur alongside depression, such as obsessive–compulsive disorder, and help manage thoughts and anxiety disorders. Researchers [33] found that sertraline, fluoxetine, and particularly olanzapine are the most prescribed medications for adolescents with AN in England. Nevertheless, further electronic research is required to determine their efficacy. Additionally, it is crucial to consider the potential adverse effects of the medications, which are more prevalent among adolescents compared to adults [34].

### 2.2. Bulimia Nervosa (BN)

#### 2.2.1. Psychotherapeutic Interventions

Furthermore, it has been demonstrated that FBT is efficacious for treating adolescents diagnosed with BN, in addition to AN [35]. Nevertheless, because of minor variations in symptoms, the more common progression, and a more comprehensive comprehension of the gravity of the illness, an adjustment of the model is necessary [36]. Researchers [37] explicitly highlight the subsequent disparities: The initial goal of the intervention is to develop a healthy relationship with food and decrease the dependence on laxatives, rather than focusing on immediate weight restoration. Furthermore, the primary focus of all therapy is the collaboration with the parents rather than the one-sided assumption of responsibility on their behalf. Moreover, parental involvement in the treatment is generally minimal, but it may increase if the patient is not properly managed. Compared to adolescents with AN, those with BN tend to have higher rates of comorbidity and often hide their symptoms due to feelings of guilt and shame. As a result, therapists face greater challenges in convincing parents of the seriousness of the illness and focusing their attention on the primary goals of the intervention. Nevertheless, the researcher emphasizes that because Bulimia is psychologically conflicted with the Ego, meaning its symptoms are undesirable, individuals who suffer from it are more inclined to engage in the intervention. Researchers [38] reported that the duration of the treatment is six months. During the first phase, sessions are held weekly, followed by sessions every three weeks in the second phase, and monthly sessions in the third phase.

The abovementioned study [38] found that FBT is more effective than Enhanced CBT. Nevertheless, the patient’s condition improves immediately following the intervention and continues to improve after six months. However, there is no statistically significant difference in the patient’s condition after 12 months. The cognitive–behavioral model incorporates cognitive, emotional, and social factors; the adolescent’s developmental stage; family engagement in treatment; and motivation for transformation. Researchers [39] state that more than twenty sessions are required. Their classification consists of four distinct stages, each with its objectives: During the initial phase, the adolescent will receive psychoeducation regarding the disorder’s risk factors and actively participate in the treatment through biweekly meetings. During the second phase, it is crucial to identify and address the factors that impact the therapeutic process, typically through meetings held once or twice a week. During the third phase, the symptoms are addressed through weekly meetings spanning at least eight weeks. Lastly, in the fourth stage, there is the expansion of therapeutic advantages and readiness for forthcoming challenges.

Enhanced CBT for BN is a tailored approach for individuals with BN, as described by [40]. According to research [41], this therapy occurs in two phases over 3–12 months. In the initial phase, the patient carefully records his/her eating habits and tries to eat at regular intervals, while the therapist communicates with the patient’s friends and family. The second phase continues these practices but also focuses on identifying the underlying causes of the problem and conducting behavioral experiments to address them. One study [20] suggests that to effectively use this model with adolescents, certain modifications are necessary. These include involving parents in the therapy and incorporating exercises that accommodate the cognitive and social development typical of this age group.

#### 2.2.2. Pharmacological Therapy

Unlike AN, BN can be effectively treated with pharmacotherapy, as stated by [42]. Nevertheless, it is essential to note that the approval for administering fluoxetine, a selective serotonin reuptake inhibitor, is limited to adults by the US Food and Drug Administration [43]. Researchers [44] conducted an open-label trial of fluoxetine. They found that administering the drug to adolescents resulted in a decrease in binge-eating and purging behaviors, with minimal side effects. Nevertheless, as highlighted by the researchers above, it is essential to consider that close surveillance is necessary due to the coexistence of comorbidity with suicidal tendencies.

### 2.3. Binge-Eating Disorder (BED)

#### 2.3.1. Psychotherapeutic Interventions

According to the authors of [45], adolescents diagnosed with BED exhibit the lowest level of willingness to pursue treatment actively. This is likely attributed to the recent acknowledgment of BED as an independent psychiatric condition, as indicated by the findings of their research. Cognitive–Behavioral Therapy (CBT) is the most efficacious method for treating this ailment [46]. The model applies to BN as well, given the similarity in symptomatology [47]. Nevertheless, slight alterations are attributed to the minor distinctions between the two disorders [48]. The intervention primarily aims to provide psycho-educational information on the factors contributing to and sustaining obesity and nutritional education. It also focuses on challenging the societal ideal of thinness, promoting body acceptance, conducting behavioral experiments to confront fears, and encouraging self-observation. Although binge-eating episodes decrease, weight is not significantly reduced. Only Behavioral Therapy is effective in weight reduction, as one study [49] stated. Enhanced CBT is more efficacious in heightened comorbidity [50]. Researchers [51] indicate that although the research has primarily focused on adults, the efficacy of the treatment also extends to older adolescents.

Within the same population, binge-eating episodes decrease when utilizing α-sale control and implementing an adjusted version of Interpersonal Therapy known as Interpersonal Therapy Weight Gain [52]. Interpersonal Therapy posits that dysfunctional behavior stems from interpersonal-relationship challenges and social-skill deficiencies. Hence, within the adolescent population, individuals who experience social isolation are subjected to teasing and are, thus, at a higher risk of developing obesity in adulthood are more likely to benefit from this intervention [53]. The utilization of Interpersonal Weight Gain Therapy in adolescents who are at risk of obesity serves the purpose of averting excessive weight gain. This therapy has been proven effective even one year after the completion of treatment, as demonstrated by [52].

#### 2.3.2. Pharmacological Therapy

The Food and Drug Administration in the US approved dexamphetamine di-mesylate (Vyvanse) in 2015 as the sole treatment for adults with moderate-to-severe BEDs [54]. In 2007, the same substance received approval for treating Attention Deficit Hyperactivity Disorder (ADHD) in children [55]. ADHD and BED often coexist, and there are shared mechanisms related to impulsivity that are observed in both disorders [56]. Nevertheless, when comorbidity is present, medication should be administered with caution [57]. Before 2019, there was a lack of significant research conducted on adolescents [58].

## 3. Materials and Methods

### 3.1. Aim

This systematic review aims to analyze the impact of family parameters on adolescents with eating disorders, while also exploring suitable interventions, such as psychotherapeutic approaches, the involvement of the family in these approaches, and pharmacological treatment. No other systematic review appears to have examined the integrated approach. The forthcoming investigation addressed the following research questions:

[RQ1] What are the familial factors that influence adolescents with eating disorders? and how?
[RQ2] What role does the family play in treating eating disorders in adolescents?
[RQ3] Which psychotherapeutic modalities are employed as coping mechanisms for eating disorders in adolescents?
[RQ4] What pharmacological intervention is employed as a therapeutic approach for managing eating disorders in adolescents?

The methodology employed in this systematic review adhered to the guidelines outlined in the Preferred Reporting Items for Systematic Reviews and Meta-Analyses by the PRISMA Statement [59].

### 3.2. Sample

**Figure 1:**
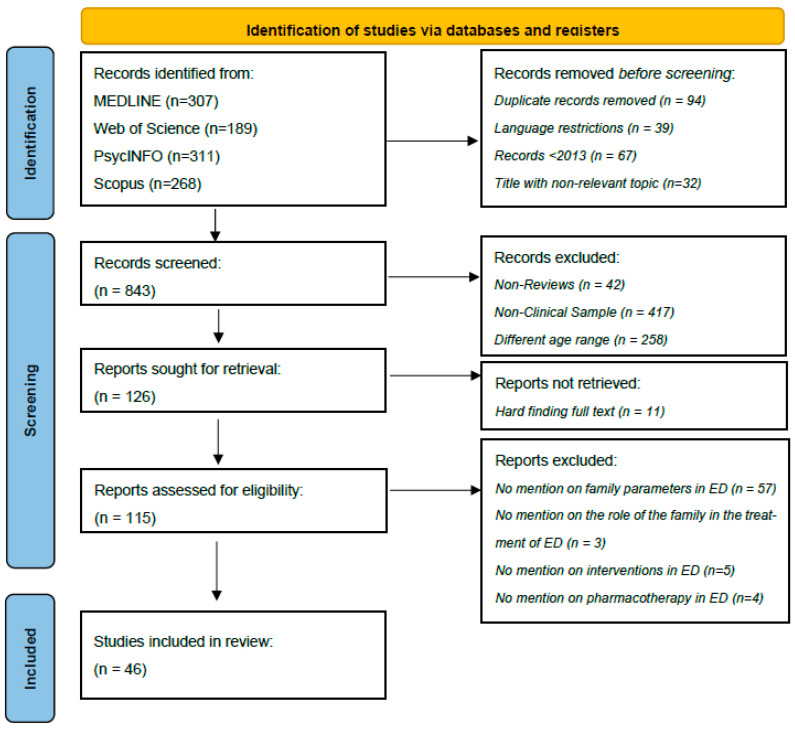
Flowchart of PRISMA methodology.

The systematic review included a total of 4794 adolescents diagnosed with AN, BN, or BED. Among them, 94% were female, and 6% were male, with an average age of 15.41 years. Additionally, 1187 adolescents were the control group, matched for sex and age. The study also involved 1563 parents, consisting of 742 mothers, 531 fathers, and 290 parents whose gender was not specified. Furthermore, 1809 siblings and 11 other relatives were included in the review. The review did not include findings related to eating disorders that were not the focus of the study, as well as any atypical variations in the eating disorders being investigated. Out of the total, 46 articles were chosen, with one being a meta-analytic review of 26 studies (Figure 1). Therefore, a total of 62 electronic surveys were examined. Out of the 8 participants, the overall average age could not be determined for 1 participant’s gender across disorders, 10 participants’ overall average age, and 15 participants’ gender in each disorder. Hence, the results were obtained by deriving them from the remaining data. The findings above pertain to AN, BN, and BED. The mean ages for these disorders are 15.09, 16.59, and 14.7, respectively.

### 3.3. Inclusion Criteria

The compilation of surveys was conducted according to the following criteria: (1) articles published in scholarly journals; (2) accessibility of articles in the English language; (3) population of individuals in the age range of 10–19 years, as defined by the World Health Organization in 2017; (4) although articles that included a non-clinical sample were considered (only as a control group), this study focused on adolescents from a clinical population, and the family members who participated in the research were selected from a non-clinical population; (5) research articles examining the impact of family factors on adolescents with eating disorders; (6) articles investigating the correlation between family dynamics and the treatment of adolescents with eating disorders; (7) articles examining psychotherapeutic methodologies in treating adolescents with eating disorders; and (8) articles exploring pharmacological interventions for adolescents with eating disorders.

### 3.4. Searching Strategy

The research inquiry was performed using the PubMed, PsycINFO, and Scopus databases, with the specified timeframe of 2013–2023. To identify appropriate publications based on selection criteria 6–9, the search engines were utilized with the following keywords: “Eating Disorders” or “Anorexia Nervosa” or “Bulimia Nervosa” or “Binge Eating Disorder” and “Adolescents”, combined with the terms “Family”, “Parenting”, “Risk Factors”, “Etiological Factors”, “Heredity”, “Intervention”, “Treatment”, or “Pharmacotherapy”.

### 3.5. Searching Procedure, Selection, and Clustering

As previously stated, the databases mentioned above were utilized for inputting the keywords. Consequently, English-language records that did not pertain to adolescents, including various age groups and non-clinical populations, were unavailable. Additionally, articles that were not published in academic journals were excluded. Each article was evaluated by examining the title (and, in thirty-two cases, the abstract and the entire article) and eliminating those that did not correspond to the search terms. Subsequently, any instances that occurred more than once were promptly eliminated, while the remaining instances were classified into the following categories: “family and eating disorders in adolescents”, “psychotherapeutic approaches and eating disorders in adolescents”, and “pharmacotherapy and eating disorders in adolescents”. The selection process is outlined in Table 1, which uses the PRISMA flowchart (Figure 1). Additionally, the review was not registered in PROSPERO.

## 4. Results

Of the 843 articles initially examined, only 46 met the selection criteria and were ultimately included in the systematic review. Out of the total, 6 studies utilized a cross-sectional research design, 4 were explanatory studies, 3 were explanatory studies that also employed secondary research methods, 4 were part of a longitudinal design, 2 were case–control studies, 5 were cohort studies, 2 were series studies, 14 were randomized controlled trials (RCTs), 1 was a non-randomized controlled trial, 2 were open trials, 1 was a naturalistic study, 1 was a correlational study, and 1 was a meta-analytic review. In addition, the geographical origins of the individuals were: 6 from Italy, 17 from the USA, 4 from the United Kingdom, 4 from Canada, 4 from Germany, 8 from Australia, and 1 each from Sweden, Brazil, Spain, and the Netherlands.

The sample predominantly comprised women, accounting for 94% of the total. Out of the 27 articles where results could be extracted, 11 had a complete representation, while the remaining had a representation exceeding 80%. According to the epidemiological data, the female sex has a significantly higher ratio than males in each disorder (AN: 94%, BN: 98%, and BED: 90%). AN was analyzed in 29 articles, BN in 12 articles, and BED in 7 articles. This fact demonstrates the extensive research conducted on each eating disorder, especially BED, which has recently been acknowledged as a distinct mental illness. Regarding familial involvement, parents were significantly more engaged, as evidenced by their mention in 15 articles, compared to siblings (mentioned in 5 articles) and other relatives (mentioned in 2 articles).

Out of the 46 articles included in the systematic review, 9 specifically examined the impact of family factors on adolescents diagnosed with the three eating disorders above. No research meeting the selection criteria and investigating the impact of heredity on the manifestation of disorders was identified during the search process. The review focused on the interventions employed by specialists in the adolescent clinical population, with 28 articles remaining for examination. Psychotherapeutic methods were examined in 26 cases, while pharmacological treatment was examined in 2. Two ubiquitous characteristics of all articles are the inclusion of measurement scales in the methodology and the presentation of the main findings.

Nine articles were examined to explore the impact of family parameters on adolescents with eating disorders, as previously stated. To be more precise, three of the studies focused on the functionality of the family system, three examined teenagers’ satisfaction with the family system, two explored parents’ comments toward their children, and one investigated the role of food in family dynamics.

The findings of the initial category demonstrate a notable degree of uniformity, and three articles documented dysfunctions within the families of adolescents diagnosed with AN. One study revealed that the functioning was significantly problematic in 7.2% of the families. Moreover, this small percentage showed a correlation with the more severe psychopathological clinical presentation of the adolescents [78]. The previous article discussed the prevalence of low coordination, unexpressed parental conflict, and lack of cooperation among the families involved in the study [90]. The final characteristic influences the manifestation of psychopathology associated with eating disorders [102]. A statistically significant correlation was found between the interaction of adolescents and their parents and their BMI [99]. The third article discovered a reciprocal relationship between AN and family functioning [70]. The psychopathological profile of teenage girls with this disorder and the parents’ risk of developing psychopathology were found to influence family functioning [97]. This relationship was observed in both BN and BED. Furthermore, the research above reveals that adolescents have distinct perceptions of family functioning about their parents across all three of the disorders examined in the systematic review [72]. We also employed the AMSTAR-2 tool to assess the methodological quality of the systematic review. AMSTAR-2 evaluates systematic reviews across 16 domains, with 7 being critical for robust reviews. The review adequately addressed all critical domains, demonstrating a strong methodological foundation (Table 2).

In the second category, there is a noticeable lack of consistency among the findings of the investigations. One article highlighted that adolescents with AN exhibited a diminished level of contentment, which was associated with their belief that their families possess lower levels of communication, adaptability, and unity, as well as greater distance, compared to families in the non-clinical population [101]. Similarly, the mothers also reported higher levels of perceived inflexibility than their children. Furthermore, an additional article corroborated the diminished level of contentment [63]. It was contended that this finding was contradictory, as the majority of adolescents with AN and BN, along with their parents, reported the presence of communication, adaptability, and intimacy in their relationships. Simultaneously, the degrees of inflexibility, hatred, and disorder are minimal. The overall dissatisfaction stemmed from the relatively low percentage of participants who reported inadequate communication within their families; however, this variation was minimal [67]. In addition, the rates were further reduced in cases where there was comorbidity with depression [73]. Contrary to the findings of previous studies, the third article revealed that adolescents with AN did not report lower satisfaction with the family system compared to individuals without this disorder [79]. Significant statistical differences were observed solely within the adolescent population, specifically in education and social interactions.

The third category pertained to BN and BED. Parents’ critical remarks characterize the former, while the latter involves parents engaging in weight teasing. In both instances, the behaviors above were more frequently observed in the parents of adolescents afflicted with specific ailments compared to individuals with other mental disorders or non-clinical groups [88]. The parents’ criticism identified differences among the families. Fathers from intact families exhibited more incredible warmth and less criticism, as did mothers, albeit with a lower frequency of positive remarks. In addition, mothers (irrespective of their marital status) exhibited reduced affection but also decreased emotional overinvolvement in the presence of the brothers [77]. Regarding weight, the only noteworthy impact on parents was that mothers’ perceptions of their adolescent’s weight were a predictor of both stigmatizing behaviors on their part and the adolescent’s development of psychopathological characteristics associated with eating disorders [66].

An investigation was conducted on a group of teenagers diagnosed with AN and BN to analyze the significance of food within Brazilian households. The results indicate that food can be used as a method of expressing both physical and psychological abuse, as well as a tool for controlling the parent–adolescent relationship and influencing the perception of the parent’s presence [90]. In addition, when combined with cultural factors, it influences the management of emotional reactivity, serving as a point of contention between the nuclear family and three generations [86]. Grandparents, fathers, mothers, and teenagers hold divergent perspectives on the regulations and customs that younger individuals should adhere to during meals. This fact affects teenagers’ dietary habits [84].

Psychotherapeutic research has encompassed both individual and Family Therapy approaches. However, most articles focus on the efficacy of the latter approach, which primarily involves parents but may also include other family members [82]. Furthermore, a majority of articles focus on AN. As previously stated, this eating disorder is characterized by a strong correlation between the family and elevated levels of dysfunction [61]. Two studies have documented this behavioral trait, employing distinct methodologies. One contended that employing individual Psychodynamic Therapy with a focus on adolescents leads to a decrease in dysfunction and enhancement in member interaction, a restriction of disorder symptoms, and a rise in BMI following a 6-month treatment period [60]. The intervention is implemented alongside pharmacological treatment to address concurrent conditions, such as depression and anxiety disorders.

Additionally, parents receive counseling to foster a solid therapeutic relationship and mutual support among family members. This counseling also focuses on identifying and addressing problems, establishing boundaries between different generations, and addressing unresolved parental conflicts [83]. Conversely, other research indicated that FBT is effective for mild cases of AN. Following the twentieth session, there was a positive correlation between disorder remission and improvements in family functioning, communication, and problem-solving ability [87].

Self-efficacy is another characteristic that is enhanced as a result of treating the same eating disorder. Two interconnected research areas were Family Therapy and the study of adolescent patients and their parents. The initial study demonstrated a positive correlation between higher levels of parental self-efficacy and increased weight gain in adolescents [89]. An unexpected discovery was that this does not hold when there is a rise in adolescents’ self-efficacy. The second study corroborates the findings of the first study. Specifically, it highlights the mediating effect of enhanced maternal self-efficacy on enhancing adolescent weight during the tenth session [93]. According to a study, FBT for AN not only helps alleviate the symptoms of comorbid disorders but also leads to a significant reduction in their prevalence. The study reported a decrease from 54% at the start of treatment to 26% at the conclusion [80]. Another study conducted within the Family-Based and Systemic Therapy framework found that the presence of comorbid disorders was a significant factor in predicting early hospitalization. Furthermore, early hospitalization was found to negatively impact weight improvement. FBT demonstrates superior efficacy to Systemic Therapy in reducing hospitalization rates within five weeks while exhibiting no statistically significant variance in Ideal Body Weight, eating disorder symptoms, and comorbidity [8,102].

Two studies investigated the remission of AN in adolescents. Based on the initial findings, Parent-Focused Therapy demonstrates greater effectiveness than FBT. Nevertheless, the distinction between them is substantial solely during the conclusion of the treatment (remission rate: 22% and 43%, respectively), but not following six or twelve months [80]. During the initial six months, there was a reduction in dietary limitations, rituals, and food-related fixations, while the patient’s expectations and perception of the treatment’s suitability showed an increase. In the latter half, anxieties regarding body morphology and the clinical manifestations of the ailment were likewise diminished [89].

Moreover, FBT demonstrates greater efficacy in addressing severe manifestations of obsessive–compulsive disorder and cases characterized by a prolonged duration of illness. This conclusion was derived from a reassessment conducted after 12 months. Parent-Focused Therapy operates oppositely [83]. Another study demonstrated a decrease in OCD symptoms when FBT was combined with either Art Therapy or Cognitive Rehabilitation Therapy. These combinations enhance the suitability of psychotherapy, the patient’s anticipations, and the therapeutic bond [82].

Unlike the initial study, the second study, which also focused on disorder remission, found that FBT significantly enhanced the likelihood of remission one year after the intervention concluded. Nevertheless, the evaluation in this specific research was conducted on separate interventions [102]. Based on the findings of the same study, the latter option is the most suitable outpatient intervention, as the participants exhibit the highest level of satisfaction.

One study stood out among the others. Although the research subject remained unchanged, the emphasis shifted to a different facet of Family Therapy. The study focused on analyzing the specific strategies employed by parents of adolescents with AN during mealtime as part of their treatment. The findings indicated a higher frequency of direct messages regarding food, indirect messages regarding food, messages about the body, information or choices regarding food, and independent comments [103]. Most mothers primarily employed the third strategy, followed by the first, while most fathers primarily utilized the first strategy, followed by the fifth. Adolescents who received direct and indirect food messages, fathers’ information, and mothers’ body messages were more likely to consume small amounts of food. Except for information provision and fathers’ food-independent comments, all strategies negatively correlated with positive adolescent comments. Regarding the food, the number of positive comments is significantly lower than that of negative comments [104].

A study was conducted involving families of adolescents with AN to determine the impact of fathers on the patients. Families were assigned to either FBT or Parent-Focused Therapy, with no statistically significant difference observed between the two approaches. It was found that increased father involvement led to a decrease in their children’s symptoms at the treatment’s conclusion and an increase in the Index Body Mass and Recession Rates [76]. Fathers who were divorced had a lower level of participation. Furthermore, it was discovered that the mothers consistently attended the treatment sessions, while the fathers gradually decreased their attendance, and the siblings experienced a significant decline in their participation rate. Regarding siblings, a separate study on AN in adolescents demonstrated that while adolescents expressed a desire to engage in treatment and gain a deeper understanding of the disorder, parents and patients did not share this sentiment. Reasons for this included concern, guilt, and the desire to protect siblings from imitating behaviors [101].

In contrast to the findings of the studies above, a single study asserted that FBT was insufficient for the parents of adolescents with AN. During the initial stage, anxiety was alleviated by offering pertinent instructions, although the impact was short-lived. Despite attempting to evade accountability by employing persuasive language and attributing the causes of the illness to external factors, parents exhibited heightened feelings of culpability at the onset of treatment. The origin of this sensation stems from the extent of the perceived impact on the manifestation of the illness, the challenge in differentiating between its onset or recurrence, and the burden of responsibility regarding dietary choices at the start of the treatment. Additionally, there is a belief that (a) adolescents have no say in the matter throughout its duration and (b) the intervention primarily concentrates on body weight. Consequently, ultimately, this leads to the generation of frustration and an escalation of concerns regarding the welfare of the children.

Out of all the articles that examined psychotherapeutic treatments for AN, three specifically focused on CBT (or its variations). In the initial study, the model was implemented without any alterations, and a subset of adolescent participants had prior involvement in FBT. Nevertheless, the outcomes exhibited a resemblance and were associated with the constraint of clinical harm, an increase in body mass, and a decline in disorder-associated behaviors. The previous two items were linked to the length of time the eating disorder persisted. Enhanced CBT was utilized in the second instance. The results mirror those of the initial treatment, with an added decrease in overall psychopathology, nutritional worries, and concerns about body shape and weight. The persistence of changes was also observed twenty weeks after the completion of treatment. The third study employed an intensive family exposure approach rooted in CBT. The findings from both prior studies remain applicable in this scenario, with the inclusion of a decrease in obsessions and rituals, depressive symptoms, and anxiety, as well as the frequency of body checking [94].

Five articles were examined to investigate BN in adolescents, and Family Therapy was utilized as the intervention in three of these studies. FBT was provided in conjunction with Dialectical Behavior Therapy in the hospital setting as part of one of the three studies. The other two subjects compared CBT specifically tailored for adolescents and supportive psychotherapy. The incorporation of Dialectical Behavior Therapy proved to be efficacious as it resulted in a decrease in objective binge-eating episodes, self-induced vomiting, weight concerns, and the frequency of covert food consumption among adolescents [87]. Additionally, their clinical presentation and emotional-regulation skills exhibited improvement following the intervention. The parents of the patients with condition A also demonstrated positive outcomes, as they experienced an increase in their self-efficacy levels. FBT demonstrates greater efficacy in a specific aspect than adolescent-tailored CBT. Specifically, individuals who actively engaged in FBT and possessed higher levels of motivation to attain change experienced a more significant reduction in their eating disorder-related behaviors. Nevertheless, in both instances, there was an enhancement in the operation of the cognitive processes. When comparing Supportive Psychotherapy to FBT, the latter is superior only in specific circumstances, namely when the patient is younger and has a higher frequency of laxative use [80].

Out of the two remaining studies, one focused on BN and utilized CBT designed explicitly for this disorder, while comparing it to Psychodynamic Therapy. The other study employed Enhanced CBT. The initial study found no statistically significant disparity among the psychotherapeutic methodologies. In CBT, participants experienced a slightly more significant reduction in binge eating and laxative use, whereas in Psychodynamic Therapy, there was a focus on addressing eating concerns. An observed characteristic was an enhancement in both overall and Bulimia-related mental disorders. Furthermore, all modifications remained consistent for one year following the completion of the treatment. The second study found that Enhanced CBT was effective in decreasing instances of binge eating, vomiting, and purging behaviors, as well as reducing psychopathological features and comorbid disorders [75].

The findings of the most recent study are also relevant to BED. The sample consisted of adolescents diagnosed with eating disorders, and the findings were shared among both groups. Two additional articles analyzed the identical clinical population. One study prioritized the examination of the disorder itself, while the other focused on analyzing the dynamics of the therapeutic relationship during the intervention. In one case, Enhanced CBT was utilized, while the classical model of CBT was employed in the other case. In the initial study, participants in the intervention group experienced a decrease in the frequency of binge-eating episodes and a higher rate of disorder remission compared to those in the control group who were on a waiting list [91].

Nevertheless, there were no discernible disparities in quality of life, comorbid depressive symptoms, self-esteem, and BMI (with a notable improvement observed upon reassessment). The second study revealed two negative correlations and one positive correlation. The patient’s expectations of treatment and the therapeutic alliance were negatively correlated with the number of binge-eating episodes and loss of control over the attachment relationship with the therapist. Conversely, a strong positive relationship was found between the therapeutic alliance and the attachment relationship with the therapist [99]. This has implications for the internal validity of the psychotherapeutic approach, mainly when these relationships are highly intense.

Regarding pharmacological treatment, two studies were exclusively dedicated to this topic. One of the studies focused on adolescents diagnosed with AN, BN, or BED, while the other specifically targeted adolescents with AN. Both share that the US Food and Drug Administration has not approved any medication for use by individuals of such a young age. The initial study centers on examining the drugs that are commonly utilized by adolescent patients, even though they lack official approval. Meanwhile, the subsequent study focuses on assessing the efficacy of olanzapine in this population. Approximately 33% of the medications consumed by American adolescents are psychotropic. This usage is more prevalent among non-Hispanic White individuals who have been diagnosed with an eating disorder and have received both inpatient and outpatient treatment in the past. Primarily, antidepressants are prescribed for individuals with a prolonged duration of the disorder and prior treatment.

Additionally, atypical antipsychotics are recommended for those who have experienced previous hospitalization, while their use is less common among individuals with higher family incomes. However, the prevalence of BED was only 1.2% in the sample. In the case of AN, the second study demonstrated that the administration of olanzapine resulted in weight gain for both the experimental group and the control group (which did not receive the drug). Nevertheless, the outcomes were more favorable in the initial scenario, with a weight loss of 12.22 kg over ten weeks, compared to the subsequent scenario, which resulted in a weight loss of 7.55 kg over the same duration. Furthermore, all participants experienced a decrease in their levels of depression and anxiety, reported a decrease in symptoms of eating disorders, enhanced their body satisfaction, and diminished their desire to lose weight. Both groups achieved similar results in this case. Notably, 31.8% of the participants encountered mild side effects and withdrew from the research [72,79].

Within the scope of the studies, two articles specifically examined the impact of treatment on perfectionism. Both interventions specifically targeted adolescents suffering from AN. However, one employed FBT in contrast to Systemic Therapy, whereas the other utilized a combination of FBT and CBT to address perfectionism. The initial article found that maladaptive perfectionism in adolescents was a reliable indicator of psychopathology related to ABP at both the 6- and 12-month follow-up assessments. Furthermore, the rates of perfectionism were comparable, irrespective of the psychotherapeutic methodology.

Conversely, the second article demonstrated a decrease in perfectionism from its initial levels after implementing CBT. Nevertheless, the socially prescribed perfectionism remained uncontrolled. Moreover, the implementation of FBT resulted in a broader decrease, which was linked to decreased rates of eating-disorder psychopathology. This reduction was observed compared to the stage where only CBT was utilized to address perfectionism [75].

Based on the information provided, it can be inferred that personality traits are likely to be commonly linked to eating disorders and their treatment in adolescents. This study conducted a meta-analytic review on a sample of adolescents diagnosed with AN, BN, or BED to examine the association between these disorders. The research revealed disparities in the personality traits of individuals afflicted with the illness compared to their healthy counterparts. More specifically, there is an increase in negative efficacy and distancing and a decrease in mindfulness. Regarding the latter, the therapist finds it highly beneficial to be aware of the connection with eating disorders, as it aids in comprehending the importance of prioritizing emotional regulation during treatment.

In Table 3, the initial categorization of all 46 articles is presented in the systematic review and information on their geographical origin, research design, and sample characteristics. The elements and the primary findings of each study are presented in Table 1, which provides a second categorization of all the systematic review articles (*n* = 46).

### Main Findings

Nine articles examined the impact of family parameters on adolescents with eating disorders, as previously stated. To be more precise, three of the studies focused on the functionality of the family system, three examined teenagers’ satisfaction with it, two explored parents’ comments to children, and one investigated the role of food in family dynamics.

The findings of the initial category demonstrate a notable degree of uniformity, as all three articles documented dysfunctions within the families of adolescents diagnosed with AN. One study highlighted that the functioning was significantly problematic in 7.2% of the families. Moreover, within this small percentage, there was a correlation observed between the more severe clinical picture of the adolescents and their psychopathological tendencies. The previous article discussed the prevalence of low coordination, unexpressed parental conflict, and lack of cooperation among the families involved in the study. The final characteristic influences the manifestation of psychopathology associated with eating disorders. A statistically significant correlation was found between the interaction of adolescents and their parents and their BMI. The third article revealed a reciprocal relationship between AN and family functioning. The psychopathological profile of teenage girls with this disorder and the parents’ risk of developing psychopathology were found to influence family functioning. This influence was observed in both BN and BED. Furthermore, the research above reveals that adolescents hold distinct perceptions of family functioning compared to their parents in all three disorders under investigation in the systematic review [92,97].

Within the second category, there is a noticeable lack of uniformity among the research findings. One article highlighted that adolescent with AN exhibited a diminished level of contentment, which was associated with their belief that their families are less communicative, adaptable, and united, as well as more distant, compared to the families of individuals without clinical conditions. Similarly, the mothers also reported higher levels of perceived inflexibility than their children. Furthermore, an additional article substantiated the diminished level of contentment. It was contended that this finding was contradictory, as most adolescents with AN and BN, as well as their parents, reported the presence of communication, flexibility, and closeness in their relationships. Simultaneously, the degrees of inflexibility, animosity, and disorder are minimal. The overall dissatisfaction stemmed from the relatively lower yet somewhat inconsistent percentage of participants who reported inadequate communication within their families [104].

Furthermore, the rates were further reduced in cases where there was comorbidity with depression. Contrary to the results of previous studies, the third article revealed that adolescents with AN did not express lower levels of satisfaction with the family system compared to individuals without this disorder. Significant statistical disparities were observed solely among the adolescent population, specifically in education and social interactions [92].

The third category pertained to BN and BED. Parents’ critical remarks characterize the former, while the latter involves parents engaging in weight teasing. Both scenarios exhibited a higher prevalence of the abovementioned behaviors among parents of teenagers afflicted with specific ailments compared to individuals with other mental disorders or those without clinical conditions. The parents’ criticism identified differences among the families. Fathers from intact families exhibited more incredible warmth and lower levels of criticism, as did mothers, who displayed a reduced frequency of positive remarks. In addition, regardless of their marital status, mothers exhibited reduced levels of affection and emotional overinvolvement in the presence of siblings. Regarding weight, the only notable impact on parents was that mothers’ perceptions of their adolescent’s weight were a predictor of both stigmatizing behaviors on their part and the adolescent’s development of psychopathological symptoms associated with eating disorders [97].

A study conducted among adolescents diagnosed with AN and BN investigated the influence of food within Brazilian families. Based on the findings, food serves as a method for expressing both physical and psychological mistreatment, as well as a tool for exerting control over the parent–adolescent relationship and influencing the perception of the parent’s presence. In addition, when combined with cultural factors, it impacts the management of emotional reactivity, serving as a source of conflict between the nuclear family and three generations. Grandparents, fathers, mothers, and teenagers hold divergent viewpoints regarding the regulations and customs that younger individuals should adhere to during meals. This fact influences the behavior of the latter about their dietary choices [101].

Psychotherapeutic research has encompassed both individual- and family-therapy approaches. However, most articles focus on the efficacy of the latter approach, which primarily involves parents but may also include other family members. Furthermore, most articles focus on AN. As previously stated, this eating disorder is characterized by a strong correlation between dysfunctional family dynamics and its occurrence. Two studies have documented this behavioral trait, employing distinct methodologies. One individual posited that implementing individual Psychodynamic Therapy with a focus on adolescents can decrease dysfunction and enhance member interaction [86].

Moreover, it can limit disorder symptoms and increase BMI over a six-month treatment period. The intervention is implemented alongside pharmacological treatment to address coexisting conditions, such as depression and anxiety disorders. Additionally, parents receive counseling to foster a solid therapeutic relationship and mutual support among family members. This counseling aims to enhance problem recognition, establish generational boundaries, and address unresolved parental conflicts. Conversely, the other research indicated the efficacy of FBT for mild cases of AN. After twenty sessions, there was a positive correlation between disorder remission and improved family functioning, communication, and problem-solving ability [92].

Self-efficacy is another characteristic that is enhanced because of treating the same eating disorder. Two interconnected research studies focused on Family Therapy and examining adolescent patients and their parents as shared subjects. The initial study demonstrated a positive correlation between higher levels of parental self-efficacy and increased weight gain in adolescents. An unexpected discovery was that this does not hold when there is a rise in teenagers’ self-efficacy. The second study corroborates the findings of the first study. Specifically, it highlights the mediating effect of enhanced maternal self-efficacy on enhancing adolescent weight during the tenth session. According to a study, FBT for AN not only aids in alleviating the symptoms of comorbid disorders but also results in a significant decrease in their prevalence. The study reported a reduction from 54% at the start of treatment to 26% at the conclusion. Another study conducted in Family-Based and Systemic Therapy found that having comorbid disorders was a predictor of early hospitalization, and being hospitalized early negatively affected weight improvement. FBT demonstrates superior efficacy compared to Systemic Therapy in reducing hospitalization rates within a five-week timeframe, while exhibiting no statistically significant variance in Ideal Body Weight, eating disorder symptoms, and severity of comorbid mental disorders after treatment or six months after that [95].

Two studies investigated the remission of AN in adolescents. Based on the initial findings, Parent-Focused Therapy demonstrates greater efficacy than FBT. Nevertheless, the disparity between the two groups is only notable in remission rate, with percentages of 22% and 43% observed after treatment. However, this discrepancy is not observed after six or twelve months. During the initial six months, there was a reduction in dietary limitations, rituals, and fixations related to food, while the patient’s anticipation of the treatment and its suitability increased. In the latter portion, anxieties regarding physique and pathological manifestations of the illness were likewise diminished [96].

Moreover, FBT exhibits greater efficacy in addressing severe symptoms of obsessive–compulsive disorder and cases with a prolonged duration of illness. This conclusion was derived from a reassessment conducted at the 12-month mark. Parent-Focused Therapy operates oppositely. An additional study demonstrated a decrease in symptoms of OCD when FBT was combined with either Art Therapy or Cognitive Rehabilitation Therapy. The combinations mentioned play a role in determining the suitability of psychotherapy, the patient’s anticipated outcomes, and the therapeutic relationship.

Unlike the initial study, the second study, which also focused on disorder remission, found that FBT significantly enhanced the likelihood of remission one year after the intervention concluded. Nevertheless, the evaluation in this specific study was conducted on individual therapies. Based on the findings of the same study, the latter option is considered the most suitable outpatient intervention, as the participants demonstrate the highest level of satisfaction [93].

One study stood out among the others. Although the research subject remained unchanged, the emphasis shifted to a different facet of Family Therapy. The study focused on analyzing the specific strategies employed by the parents of adolescents with AN during mealtime as part of their treatment. The findings indicated a higher frequency of direct messages about food, indirect messages about food, messages concerning the body, information or choices regarding food, and independent comments. Most mothers primarily employed the third strategy, followed by the first strategy, while most fathers primarily utilized the first strategy, followed by the fifth strategy [90]. The study found a positive correlation between direct and indirect food messages, fathers’ information, and mothers’ body messages and the tendency of adolescents to consume small amounts of food. Except for information provision and fathers’ food-independent comments, all strategies negatively correlated with positive adolescent comments. Regarding the food, there are significantly fewer positive comments than negative ones.

A study involving families of adolescents with AN revealed the impact of fathers on the patients. Participants were assigned to either FBT or Parent-Focused Therapy, with no statistically significant difference observed between the two approaches. Notably, increased father involvement was associated with a decrease in the child’s symptoms after treatment and an increase in the Index Body Mass and Recession Rates. Fathers who were divorced had a lower level of participation. Furthermore, it was discovered that the mothers consistently attended the treatment sessions, while the fathers gradually decreased their attendance, and the siblings experienced a significant decline in their participation rate. Regarding the latter, a separate study on AN in adolescents demonstrated that, although adolescents expressed a desire to engage in treatment and gain a deeper understanding of the disorder, the same cannot be said for parents and patients [89]. Concern, guilt, and the desire to prevent them from imitating behaviors are the reasons.

In contrast to the conclusions drawn from the studies above, a single study asserted that FBT was insufficient for parents of adolescents with AN. Initially, anxiety was alleviated by providing pertinent instructions; however, the impact was transient. Despite attempting to evade accountability by employing persuasive language and attributing the causes of the illness to external factors, parents exhibited heightened feelings of culpability at the onset of treatment. The origin of this emotion stems from the extent of the perceived impact on the manifestation of the illness, the challenge of differentiating between its onset or recurrence, and the burden of responsibility regarding dietary choices at the start of the treatment. Additionally, there is a belief that (a) adolescents have no say in the matter throughout its duration, and (b) the intervention primarily concentrates on weight-related issues. Consequently, ultimately, this leads to frustration and an escalation of concerns regarding the well-being of the children [75].

Out of all the articles that examined psychotherapeutic treatments for AN, three specifically focused on CBT or its variations. In the initial study, the model was implemented without any alterations, and a subset of adolescent participants had prior involvement in FBT. Nevertheless, the outcomes correlated closely with diminished clinical debilitation, increased body mass, and alterations in disorder-associated behaviors. The previous two items were linked to the length of time that the eating disorder persisted. Enhanced CBT was implemented in the second instance. The results mirror the initial treatment’s results, with the added benefit of decreased overall psychopathology, diminished nutritional worries, and reduced concerns about body shape and weight. Twenty weeks after the treatment’s conclusion, the changes’ stability was also observed. The third study employed an intensive family exposure approach rooted in CBT [78]. The current situation aligns with the findings from the previous surveys, highlighting a decrease in obsessions and rituals, depressive symptoms, anxiety, and frequency of body control.

Five articles were examined to investigate BN in adolescents, with three of them utilizing Family Therapy as the intervention. FBT was provided concurrently with Dialectical Behavior Therapy in the hospital setting as part of one of the three studies. The other two subjects compared CBT tailored for adolescents and supportive psychotherapy. The integration of Dia-Verbal Behavioral Therapy proved to be efficacious, as it resulted in a decrease in objective binge-eating episodes, self-induced vomiting, weight concerns, and the frequency of covert food consumption among adolescents. Additionally, it improved their clinical presentation and emotional regulation skills. The parents of the patients with condition A also demonstrated favorable outcomes, as they experienced an increase in their self-efficacy levels [66]. FBT demonstrates superior efficacy in a specific aspect compared to adolescent-tailored CBT. Specifically, individuals who actively engaged in FBT and exhibited higher levels of motivation to attain positive transformation experienced a more significant reduction in their eating disorder-related behaviors [86,90]. Nevertheless, in both instances, there was an enhancement in the operation of the cognitive processes. When comparing Supportive Psychotherapy and FBT, it is evident that FBT is superior, but only in specific circumstances, such as when the patient is younger and has a higher frequency of laxative use [77,79,102].

Among the two remaining studies, one investigated the use of CBT specifically designed for BN, compared to Psychodynamic Therapy. The other study focused on enhanced CBT. The initial study found no statistically significant disparity among the psychotherapeutic methodologies. In CBT, participants experienced a slightly more significant reduction in binge eating and laxative use compared to Psychodynamic Therapy, which primarily addressed eating concerns. An inherent characteristic was the enhancement of both overall and Bulimia-specific psychological disorders [92]. Furthermore, all modifications remained consistent and unchanged one year following the conclusion of the treatment. The second study found that Enhanced CBT effectively reduced binge-eating episodes, vomiting, purging behaviors, psychopathological features, and comorbid disorders [68,91].

The findings of the most recent study are also relevant to BED. The sample consisted of adolescents diagnosed with various eating disorders, and the findings were shared among both groups. Two additional articles analyzed the identical clinical population. One study concentrated on the pathology of the disorder, while the other examined the dynamics of the therapeutic relationship during the intervention. One instance involved the application of enhanced CBT, while the other utilized its traditional model. In the initial study, participants who were on a waiting list (control group) had a lower frequency of binge-eating episodes and a higher rate of disorder remission compared to the other group [64,67,75].

Nevertheless, there were no discernible disparities in quality of life, comorbid depressive symptoms, self-esteem, and BMI (with a notable improvement observed upon reassessment) [75,80,81]. The second study revealed two negative correlations and one positive correlation. The patient’s expectations of treatment and the therapeutic alliance were negatively correlated with the number of binge-eating episodes and loss of control over the attachment relationship with the therapist [63,88,89]. In contrast, a direct association was observed between the therapeutic alliance and the attachment relationship with the therapist, which impacts the internal validity of the psychotherapeutic approach, particularly at elevated levels [60,77,82].

Regarding pharmacological treatment, two studies were exclusively dedicated to this topic. One of the studies involved a sample of teenagers diagnosed with AN, BN, or BED. In contrast, the other study specifically focused on teenagers diagnosed with AN. A shared characteristic between the two is that the Food and Drug Administration has not approved any medication for individuals of such a young age. The initial study examines the prevalence of drug usage among adolescent patients, even in the absence of official approval. The subsequent study evaluates the efficacy of olanzapine specifically in this population. Psychotropic drugs account for 33% of the medications consumed by American teenagers, with a higher prevalence observed among non-Hispanic White individuals diagnosed with an eating disorder and who have received previous inpatient and outpatient treatment. Primarily, antidepressants are prescribed for individuals with a prolonged duration of the disorder and prior treatment [60,77,84].

Within the range of studies, two articles specifically examined the impact of treatment on perfectionism. To be more precise, both were targeted toward adolescents suffering from AN. However, one employed FBT in contrast to Systemic Therapy, whereas the other utilized a combination of FBT and CBT to address perfectionism. The initial article found that maladaptive perfectionism in adolescents was a reliable indicator of psychopathology related to ABP at follow-up assessments conducted at 6 and 12 months. Furthermore, the rates of perfectionism remained consistent, irrespective of the psychotherapeutic method employed [69,81].

Finally, based on the information provided, it can be inferred that personality traits are likely to be commonly linked to eating disorders and their treatment in adolescents. This study conducted a meta-analytic review, using a sample of adolescents diagnosed with AN, BN, or BED to examine the association between these disorders. The research revealed disparities in the personality traits of individuals afflicted with the illness compared to their healthy counterparts. More specifically, there is an increase in negative efficacy, a greater sense of distancing, and a decrease in mindfulness. The therapist finds it highly beneficial to understand the connection between the decrease in mindfulness and eating disorders, as it highlights the importance of prioritizing emotional regulation during treatment [66,69,71].

## 5. Discussion

Family influences on adolescents with AN, BN, and BED were examined in this systematic review. This article analyzes effective psychotherapeutic methods, focusing on family-system integration and pharmacological interventions. According to this classification, all 46 articles were divided, chosen, and used to obtain results. As mentioned in the Introduction, eating disorders, especially in adolescents, are understudied. Pharmacological treatment had the fewest studies, two.

Since the US Food and Drug Administration has not approved any medication for adolescents, there was expected to be little literature. Scientific research on family parameters (i.e., nine articles) has only begun in the last decade [106]. However, psychotherapeutic interventions for Anorexia and Bulimia have been extensively studied. It was surprising to find only five inquiries related to Bulimia. Due to the recent recognition of BED as a mental illness, there are few studies (three) on it.

In particular, the systematic review found that family dysfunction affects adolescents with AN. Some relationships are causal, while others are correlations. Reviewing the control groups revealed their differences. According to researchers [107], clinical family self-reports show significantly lower communication and cooperation than non-clinical families. Another discovery was the link between family dysfunction, AN intensity, and BMI. Conversely, other researchers [108] argue otherwise. They found no correlation between the two variables in one study and noted that the patient’s weight coefficient is very low. One study found a strong positive correlation between family dysfunction and adolescent diet.

The systematic review also showed that AN affects family functioning. The relationship is reciprocal. Adolescent psychopathology affected family functioning. According to other researchers [109], once the illness symptoms appear, parents feel powerless to help and understand their child. Thus, they feel fear and guilt. The systematic review also found that psychopathological parents harm the family system. The strongest correlation between mental disorders and consanguinity is among first-degree relatives [110,111]. Thus, if a parent has a mental disorder, his/her child is more likely to develop one. If the child develops the disorder, he/she may pass it on to his/her children. Children of bipolar, personality, or anxiety/depression sufferers are more likely to develop eating disorders [112]. Thus, parents of children with any of the above disorders are more likely to develop psychopathology. This condition causes stigma, stress, and difficulty assuming responsibilities, thus hinders family functioning [113].

Family discord directly affects family happiness. The systematic-review results were inconsistent. The international literature follows a similar pattern. There is no statistically significant difference between AN and non-clinical families, according to some studies. A study [114] found that AN patients perceive father rejection and feel a greater need to succeed to please their mothers. Dysfunctional families reduce satisfaction. Parents may also be unhappy. Mothers of adolescent girls with AN are less satisfied with their unity and connection with their spouses and children than non-clinical families [115]. Similar findings were found in [116]. However, the researchers note that adaptability, clinical, and non-clinical families all report similar levels of family system satisfaction. Thus, understanding the relationship between family dysfunction and happiness is crucial.

Similar outcomes for adolescents with BN and BED include high parental criticism and weight teasing. These behaviors harm adolescents’ mental health and food relationships. Researchers found that mothers of adolescents with BN often obsess over diet, weight, and body shape, expressing disapproval. Although less so, fathers contribute to this dynamic [117]. Additionally, mothers may impose strict diet and exercise regimens on their daughters, especially overweight ones, thus worsening the issue [118]. Parental weight teasing stigmatizes and increases BED-related psychological issues [119]. Family criticism is less effective than peer or friend teasing, which is more likely to occur [120].

Food is central to all three eating disorders. A Brazilian study found that adolescents use AN and BN to express trauma and cope with negative emotions. The effects of these disorders vary by culture. According to Chinese research, adolescents with AN feel obligated to eat because of parental obligations, family hierarchy, and perceived parental authority [121]. Different eating habits among three generations in a family also cause negative food attitudes. Another Brazilian study found that three generations with AN—grandmother, mother, and daughter—transmitted sexuality inhibitions and shame, highlighting strong family-system effects [122].

The most effective psychotherapy for AN is FBT. Psychodynamic Therapy for adolescents is also used in treatment. Disruptive patterns are reduced by these interventions, which improve body mass and family functioning. Family involvement in individual-focused interventions is supported by research. Ego-Oriented Individual Therapy, a type of Psychodynamic Therapy, increases BMI and family interaction by reducing negative communication and promoting positive communication [123].

This systematic review found that FBT improves family functioning and treatment-related disorder remission. This supports FBT’s better outcomes than individual treatments and comparability to Parent-Focused Therapy. All family members participate in FBT, but parents manage the adolescent’s treatment [124,125]. FBT improves parent self-efficacy and reduces other medical conditions besides AN [126,127]. Parents’ confidence grows when their adolescent’s eating resistance decreases, improving their overall health [128]. Parents who accept responsibility for their children’s food have higher self-efficacy and lower comorbidity, reducing anxiety and depression symptoms [129].

Due to a typically more distant relationship with their fathers before treatment, adolescents often change their behavior and attitudes faster when influenced by their fathers than their mothers. Increased closeness during treatment improves weight and reduces restrictive behaviors [130].

FBT also helps quit disorders. Weight management, treatment, and psychological support improve health and remission. Early weight gain during Family-Focused Therapy predicts AN resolution [131,132].

Increased father involvement in Family Therapy reduces teen symptoms, according to research. Fathers discontinue treatment faster than mothers, but not as quickly as siblings, who feel excluded [133,134]. The global literature suggests that including all family members in therapy is beneficial [135,136]. Fathers’ and mothers’ roles have not been extensively studied.

The final stage of FBT involves parents refeeding their children and assessing their beliefs about high-nutrition foods. When parents work together, teens are more likely to cooperate [137]. Conflicts from tense relationships may affect long-term parent–child relationships, explaining differences in engagement between divorced and married fathers [138].

FBT effectively treats AN, improving individual and family outcomes. Parental self-efficacy, comorbidity, weight management, and psychological health improve, increasing remission rates [139]. Parents and siblings can be involved in therapy to improve outcomes.

Family-oriented therapy benefits the adolescent and the family. As stated in the Introduction and shown by the systematic review, it is the most effective treatment for AN. Parents often feel responsible for shaping their child’s trajectory and viewpoint, so they think that teens cannot express themselves. Additionally, parents may be unhappy with the excessive focus on weight. Strangely, the older literature does not mention this discovery. Prior studies show that the intervention helps with weight regain, therapeutic alliance, and symptom relief [140,141].

The latest research supports the review’s findings that therapists pressure parents to focus solely on weight gain [142,143]. Thus, they feel guilty and fatigued, doubt the expert’s therapeutic bond, and often disengage before the intervention ends.

Few articles evaluated CBT or its adaptations for AN adolescent. Compared to enhanced CBT, intensive family exposure had slightly greater effects. Classic CBT yielded additional findings. Their differences were so small that conclusions were impossible. Incorporating the family in the first instance and cross-diagnostic methodology (adapting to each patient’s traits) in the second instance [144] may have increased effectiveness. The systematic review of the global CBT literature shows improvements in weight management and eating behaviors.

There are also references to family involvement improving communication [145]. The results from Enhanced CBT align with those reported in the Section 4. In their study, [146], AN-diagnosed adolescents improved in weight and clinical condition. Additionally, eating disorder-related psychological issues, nutrition, body shape, and weight concerns decreased. Except for one study mentioned in the Section 4, family-based CBT for adolescents with AN has not been extensively studied. Its efficacy should not be extrapolated to other cases because it is likely a highly specialized variant of CBT.

Family-Focused Therapy, Psychodynamic Therapy, and CBT can treat adolescent BN. Initial studies showed that parental involvement reduced binge eating and purging [147]. After bibliographic research, a group of researchers [148] confirmed the discovery, while another group [149] conducted the first randomized controlled trial in adolescents. The latter group compared Supportive Psychotherapy, as did an e-research study in the Section 4. Although both psychotherapeutic approaches were effective, Family Therapy was significantly better in this case. CBT was also proven to be effective by research. A customized approach that addresses adolescents’ needs reduces the risk of concurrent disorders and abnormal psychological traits; reduces illness symptoms; and reduces concerns about diet, body image, and weight [150,151,152]. Most of these discoveries came from systematic reviews. Researchers found CBT (Bulimia-specific) and Psychodynamic Therapy to be effective [153].

BED in adolescents is treated effectively with CBT and Enhanced CBT. The 2013 recognition of BED as a mental illness limited research discovery before 2015, when the systematic review began. CBT reduced binge eating and other disorders and psychological issues, according to a study [154]. The model works for all disorders and can be adapted for adolescents, but no study has examined Enhanced CBT for BED. They usually involve Eating Disorders Not Otherwise Specified.

BED has not worked in Family Therapy, unlike the other two eating disorders. The systematic review also found that AN had more initial psychotherapeutic-approach research than BN, which was nearly equal. Prognosis and therapeutic-objective discrepancies may explain this. As stated in the Introduction, AN has a worse prognosis than BN and BED. Preventing mortality in the first scenario is more important than in the others. FBT promotes weight gain and electronically monitors adolescent food intake to achieve this goal. Instead, CBT educates about the illness and reorganizes cognitive functions.

In addition to psychotherapy, adolescents with AN, BN, or BED were treated pharmacologically. Since no FDA-approved drug has been approved for this group, the results focused on which drugs are effective or commonly used by adolescents. Antidepressants and atypical antipsychotics were prescribed for AN, and olanzapine worked [155]. Weight and body-shape concerns and eating phobias are relieved by olanzapine [156]. It also reduces psychopathology, increases body mass and medical response, and improves sleep [157].

However, e-survey efficacy is disputed. Some researchers [158] claim that placebo and treatment have similar effects. They found similar increases in appetite, mobility, disorder-related psychopathology reduction, and weight gain. Treatment only improved glucose and insulin metabolism in the experimental group. Using olanzapine as a supplement to psychotherapy for adolescents was essential in all studies.

In a literature review [159], antidepressants and antipsychotics are recommended for treating AN, BN, and Eating Disorders Not Otherwise Specified in children and adolescents, but BED is not mentioned. Previous BED studies focused on obese adolescents and sibutramine, an appetite suppressant banned in many countries [160,161,162,163]. The systematic review’s BED study sample size is too small to draw conclusions.

Psychotherapy can affect eating disorder-related personality traits. Perfectionism is common in AN teens. Combining CBT for perfectionism and Family-Focused Therapy is more effective than Family Therapy alone [164]. FBT did not change perfectionism in adolescent girls with AN after 18 and 36 months, according to some research. They recommend combining CBT with other therapies [37]. The review shows personality differences between eating-disordered adolescents and controls, affecting treatment [165]. However, personality traits and BED have not been extensively studied, nor has adolescent personality affected treatment outcomes for each eating disorder [166,167,168,169].

This systematic review has limitations. Women dominated the sample and each eating disorder at first. While these data match the epidemiology, eating disorders affect a large portion of men. Therefore, future studies must thoroughly investigate this demographic. Most studies used self-reports to assess family parameters and adolescents’ circumstances before treating them. Moving toward more objective measurement scales would be beneficial. Limited survey accessibility was another constraint [170,171,172,173,174].

Since the true male/female ratio and average age are distorted, the findings cannot be generalized, especially in BN and BED. Thus, all domains need more research. One case evaluating Family-Focused Therapy in AN adolescents had enough data. The search found no adolescent studies, disproving the heritability of eating disorders. Thus, this situation requires further investigation [175,176,177,178,179,180].

The family-dynamics analysis mostly found correlations with eating disorders, not causation. Childhood-abuse studies, retrospective adult reports, and longitudinal studies are excluded. The limited research on the family’s role in adolescent eating disorders and families’ diverse cultural backgrounds make conclusions difficult [181]. Although limited, the review emphasizes the importance of family factors, especially when combined with family involvement in psychotherapeutic and pharmacological treatments. This highlights the importance of family dynamics in treatment outcomes. The family’s role in supporting adolescents extends beyond Family Therapy to individual treatments, as shown by the review. In treating adolescents with eating disorders, the family is not the only factor, but it is crucial.

FBT is the best AN psychotherapy. CBT is nearly as effective for BN and only CBT or Enhanced CBT for BED [182,183,184,185]. Olanzapine is effective for AN, but research is limited in most areas, except for FBT in adolescents with AN, so the findings cannot be generalized [186,187,188,189,190]. However, the review covers all studies and emphasizes the family’s role in effective interventions and treatment outcomes.

Finally, our findings align closely with those of a recent umbrella review [191], which synthesized evidence on the effectiveness of psychological interventions for adolescent eating disorders. Both reviews highlight FBT as the most evidence-based intervention, particularly effective for Anorexia Nervosa and Bulimia Nervosa. CBT, including Enhanced CBT, is also supported as an effective treatment, especially for BN and BED. Key outcome measures, such as improvements in eating-disorder symptoms, weight restoration, and treatment retention, were emphasized in both reviews, reinforcing the importance of these metrics in evaluating treatment success. This consistency underscores the critical role of family involvement and psychological interventions in enhancing recovery rates and reducing relapse occurrences among adolescents with eating disorders.

### Limitations

This systematic review is subject to several limitations. Firstly, the search was limited to articles published in English, potentially excluding relevant studies in other languages. Secondly, the review included only articles accessible through specific databases (PsycINFO, PubMed, and Scopus), which may have restricted the comprehensiveness of the literature search. Thirdly, the variation in research designs and methodologies across the included studies could introduce heterogeneity that impacts the comparability of results. Additionally, the reliance on self-reported data in many studies may be prone to bias. Finally, the exclusion of atypical variations in eating disorders and the focus on clinical populations may limit the generalizability of the findings to broader adolescent populations. Despite these limitations, this review provides valuable insights into the impact of family dynamics and therapeutic interventions on adolescent eating disorders.

## 6. Conclusions

Eating disorders (EDs) are serious mental illnesses that typically develop during adolescence. Atypical eating patterns, excessive preoccupation with weight or body shape, and significant medical and psychological repercussions characterize them. Family and genetic influences are essential factors in the development and persistence of various phenomena. To summarize, familial approaches are crucial in comprehending, averting, and managing eating disorders in adolescents. Incorporating family involvement and addressing familial dynamics can significantly enhance recovery rates and minimize relapses.

Furthermore, cognitive, socio-emotional, and interpersonal factors exert an influence on eating disorders in adolescents. Individuals with eating disorders exhibit impaired cognitive flexibility, a reduced ability to integrate information holistically, and challenges in understanding and responding to social and emotional cues. An in-depth understanding of eating disorders in adolescents requires careful consideration of family dynamics, relationships, and the correlation between obesity and the risk of developing an eating disorder. Efforts to prevent this should prioritize early intervention to address risk factors, like body dissatisfaction, during adolescence. It is essential to establish screening and monitoring procedures to detect individuals who are at risk. Additionally, interventions should foster positive family relationships and psychological well-being, while discouraging unhealthy weight control practices.

In conclusion, the comprehensive research examined in this paper emphasizes the significant influence that family dynamics and involvement have on the treatment of eating disorders such as AN, BN, and BED in adolescents. FBT is a highly effective intervention, especially for AN. It emphasizes the significance of parental involvement and cooperation in the treatment process. Although CBT exhibits considerable potential, additional research is needed to investigate the impact of pharmacological interventions. Given the higher number of teenage girls affected, it is crucial to consider the intricacies of family dynamics and the unique traits of adolescents when developing treatment strategies. In conclusion, the paper emphasizes the necessity of further investigation into enhancing family-based interventions. It underscores the significance of family involvement in the recovery process for adolescents struggling with eating disorders.

## Figures and Tables

**Table 1:** Main results of systematic analysis (*n* = 46).

Authors	Geographical Origin of Study	Research Design	Sample Characteristics	Main Findings
Accurso et al. (2014) [60]	USA	RCT	Total: 121 - Family-Based Treatment: unknown - Individual Adolescent Supportive Psychotherapy: unknown	- Psychological symptoms improved significantly over time for adolescents with AN, regardless of treatment type. - Weight gain was a significant predictor of improved eating disorder pathology and psychological outcomes. - The impact of weight restoration on symptoms diminished over time, highlighting the importance of early weight gain for optimal improvement.
Balottin et al. (2018) [61]	Italy	Longitudinal	24 teenagers (mean age = 14.83) →AN C = 100% 48 parents (24 mothers; 24 fathers)	Many-Facet Rasch Model (MFRM) analysis Reduction in Anorexia’s psychopathological symptoms in 54.17% (*p* < 0.05) of adolescent participants from baseline compared to 6 months later (estimated psychopathology parameter = 0.31→Time 1; estimated psychopathology parameter = −0.39→Time 2, *p* = 0.001) Log Linear Regression Improvement in the interactional abilities of adolescents, usually belonging to families, who display dysfunctional behavior patterns during the interaction phase of the child with one parent in the Lausanne Trilogue Play (0.05 < *p* < 0.07, *p* < 0.05)
Baudinet et al. (2023) [62]	UK	RCT	Total: 167	Positive caregiving experiences moderated treatment outcomes at follow-up, with participants having fewer positive experiences showing higher weight at follow-up with MFT-AN compared to FT-AN.
Byrne et al. (2015) [63]	USA	RCT	121 teenagers (average age = 14.4) →AN C = 90.9%; A = 9.1% 242 parents (121 mothers; 121 fathers)	Mixed-effects model Adolescent weight gain from baseline to end of treatment through improved parenting self-efficacy in families receiving FBT (F(1, 273.5) = 10.954, *p* < 0.001) Non-statistically significant results for the relationship between adolescent self-efficacy improvement and their weight gain (*p* < 0.10)
Ciao et al. (2015) [64]	USA	RCT	80 teenagers (average age = 16.05) C = 97.5%; A = 2.5% 37→BN	Multi-level development models The use of psychotropic drugs (*p* = 0.02) and age (*p* = 0.03) act as predictors of psychological changes. The latter (*p* = 0.02), together with the severity of purging behaviors (*p* = 0.03), mediates the results of FBT and Supportive Therapy
Craig et al. (2019) [65]	United Kingdom	RCT	54 teenagers (average age = 15.5) C = 52; A = 2 28→AN 8→BN	Intention-to-treat analysis Reduction in eating disorder-related psychopathology symptoms and clinical problems at end of treatment compared to baseline (d = 0.521/d = 0.588)
Criscuolo et al. (2020) [66]	Italy	RCT	24 teenagers (average age = 15) →AN C = 22; A = 2 48 parents (24 mothers; 24 fathers)	Chi-square tests and Wilcoxon signed-rank tests Of all the statistically significant differences regarding the functionality of families in the Lausanne Trilogue Play (Friedman’s chi-square = 52.188; *p* < 0.001), participation is the highest category (Wilcoxon signed-rank test), followed by organization (−4.219, *p* < 0.001), the concentration (−4.224, *p* < 0.001), and emotional contact (−4.249, *p* < 0.001). Correlation analysis The interaction between parents and adolescent correlates with the latter’s BMI (rho = 0.558, *p* < 0.01)
Dalle Grave et al. (2015) [67]	Italy	Cohort	68 teenagers (average age = 16.5) C = 66; A = 2 20→BN 14→BED	Intention-to-treat analysis Reduction of psychopathology symptoms related to eating disorders (t = 8.33, *p* < 0.001, d = 1.03), corresponding forms of behavior (t = 27.03, *p* = 0.001), and more general psychiatric characteristics (t = 6.27, *p* < 0.001, d = 0.66) after applying Enhanced CBT
Dalle Grave et al. (2020) [68]	Italy	Cohort	51 teenagers (average age = 15.5) →AN C = 100%	Mixed linear models Increase in BMI at the end of Enhanced CBT (β = 56.1, t = 4.32, *p* = 0.002) and its maintenance after 20 weeks (β = −29.4, t = −2.81, *p* = 0.019). The same results, but with a reduction in behavior, also apply to nutritional concerns (β = −2.9, t = −4.9, *p* < 0.001/β = 1.2, t = 2.7, *p* = 0.007), concerns regarding with weight (β = −5.4, t = −3.6, *p* < 0.001/β = 3.9, t = 3.2, *p* = 0.002) and shape (β = −4.6, t = −5.7, *p* < 0.001/β = 2.5, t = 3.9, *p* < 0.001) of the body, clinical problems (β = −29.0, t = −6.2, *p* < 0.001/β = 12.1, t = 3.3, *p* = 0.001), and general psychopathology (β = −1.6, t = −4.1, *p* = 0.001/β = 0.7, t = 2.3, *p* = 0.038)
Durfense et al. (2019) [69]	Canada	Meta-analysis	827 teenagers (mean age = 15.64) C = 96.4%; A = 3.6% 689→AN (Average age = 15.73) C = 97.2%; A = 2.8% 79→BN C = 100% 9→BED C = 100% 946 adolescents—control group (same age range and gender)	Meta-analyses Adolescents with eating disorders show statistically significant differences compared to the corresponding non-clinical population in terms of negative emotionality (g = 0.78), distancing (g = 0.69), and conscientiousness (g = −0.53)
Egbert et al. (2023) [70]	USA and Australia	Longitudinal	Total: 150 - Females: 144	- BMI improved over the time of Multi-Family Therapy (MFT) and remained improved at both the 6- and 12-month follow-ups. - Eating disorders symptomatology (measured by EDI-II) improved over the time of MFT and remained improved at both the 6- and 12-month follow-ups. - Perceived family functioning (measured by FAD) improved over the time of MFT and remained improved at both the 6- and 12-month follow-ups. - Improvement in dimensions of family functioning (roles, communication, and general family functioning) mediated the improvement of several dimensions of symptomatology (ineffectiveness, impulsivity, social insecurity, and interpersonal distrust).
Fisher and Bushlow (2015) [71]	USA	Cohort	44 teenagers (average age = 15.4) C = 38; A = 6 Diagnosis based on DSM-IV 17→AN 1→BN Diagnosis based on DSM-V 22→AN 1→BN 44 parents (34 mothers; 6 fathers)	Analysis of variance and *t*-tests Increased odds of more dissatisfaction and less communication, cohesion, and flexibility in adolescent patients and their parents who are more depressed (*p* < 0.05) Correlation analysis Statistically significant correlation of hostility (r = 0.341, *p* = 0.023) and rigidity (r = 0.460, *p* = 0.002) of adolescents and parents
Godart et al. (2022) [72]	France	RCT	Total: 60 - Treatment as Usual: 30 - Systemic Family Therapy + Treatment as Usual: 30	The intervention effects of adding Systemic Family Therapy to Treatment as Usual in adolescents with AN were as follows: - Global Outcome Categories: 60% Good/Intermediate in the Systemic Family Therapy group vs. 31% in the Treatment-as-Usual group (*p* = 0.026) - Mean BMI: significant increase in the Systemic Family Therapy group (*p* = 0.048) - Resumption of menses: 70.0% in the Systemic Family Therapy group vs. 40% in the Treatment-as-Usual group (*p* = 0.020) - Mental-state score: significant improvement in the Systemic Family Therapy group (*p* = 0.010) - Family cohesion scores: lower in the Systemic Family Therapy group (*p* = 0.040)
Gorrell et al., (2018) [73]	USA	RCT	110 teenagers (age range = 12–18 years) →BN C = 93.6%; A = 6.4%	Linear Regression Statistically significant association of motivation to change with improvement in cognitive processes at the end of FBT or adolescent-tailored CBT (F (5, 87) = 3.84, *p* = 0.003, R^2^ = 0.18) Accounting regression Statistically significant association of motivation to change with abstinence from the behaviors associated with BN at the end of the intervention, but only among adolescents who participated in Family-Based Treatment
Gorrell et al. (2020) [74]	USA	RCT	604 teenagers (mean age = 15.3) C = 90.3%; A = 9.7% 32.6%→AN 27.6%→BN 1.3%→BED	Logistic regression Comorbid psychotropic medication use is higher in non-Hispanic Whites (B = −0.47, χ2 = 4.38, *p* < 0.001) with prior inpatient care (B = 1.92, χ2 = 71.45, *p* < 0.001), prior outpatient care (B = 1.55, χ2 = 36.99, *p* < 0.001), and with an eating disorder diagnosis (BSD vs. PSA: B = −0.70, χ2 = 6.69, *p* = 0.01) Multinational logistic regression Statistically significant association of previous treatment, inpatient (B = 1.57, χ2 = 33.31, *p* < 0.001) and outpatient (B = 0.94, χ2 = 7.46, *p* = 0.006), frequency of laxative use, and longer duration of illness from eating disorder onset (B = 0.19, χ2 = 5.33, *p* = 0.02) with more antidepressant medication use. Regarding antipsychotic drugs, less use is observed when there is a higher family income (B = −0.17, χ2 = 10.17, *p* = 0.001).
Hilbert et al. (2019) [75]	Germany	RCT	73 adolescents (mean age = 15.3 ± 2.5) C = 82%; A = 18% 37→BED 36→Waiting list-group control	Intention-to-treat analysis Fewer monthly binge-eating episodes (*p* < 0.00066), greater abstinence from binge eating (*p* < 0.005), higher rates of BED symptom remission (*p* < 0.005), and lower comorbid psychopathology (*p* < 0.005) at the end of CBT compared to the control group
Hughes et al. (2017) [76]	Australia	RCT	198 families of adolescents with AN →369 parents (194 mothers; 175 fathers); 165 brothers C = 50%, A = 50%	Hierarchical Linear Regression The greater participation of fathers in the treatment is a predictive factor of the increase in the patients’ BMI (*p* = 0.039) and the decrease in the psychopathological symptoms of the disorder (*p* = 0.011) after the intervention Analysis of covariance (*p* = 0.014)
Hughes et al. (2014) [77]	United States and Australia	RCT	Total: 100	The quantitative effects of the interventions in the study are as follows: - Full remission - Conjoint Family-Based Treatment (CFT): 47% at the end of treatment, 78% at 5-year follow-up - Separated Family Therapy (SFT): 76% at the end of treatment, 90% at 5-year follow-up - Partial remission: Not specified - Changes in eating pathology, depressive symptoms, and self-esteem: Not specified
Hurst and Zimmer-Gembeck (2019) [78]	Australia	Cohort	21 teenagers (average age: 14.9) →AN C = 100%	Intention-to-treat analysis Weight gain (F(1, 20) = 76.6, *p* < 0.01) and reduction in AN symptoms (F(1, 20) = 13.8, *p* < 0.01), associated with perfectionism (F(1, 20) = 11.7, *p* < 0.01) and overcontrol (F(1, 20) = 8.6, *p* < 0.01), at the end of the intervention compared to its start
Laghi et al. (2015) [79]	Italy	Longitudinal	36 teenagers (average age = 14.86) →AN C = 100% 36 teenagers—control group (same age range, gender, and education level) 72 parents (36 mothers; 36 fathers)	Multivariate analysis of variance (MANOVA) Adolescents with AN consider that in their families there is less cohesion (F(1, 70) = 83.67, *p* < 0.001), flexibility (F(1, 70) = 36.75, *p* < 0.001), communication (F (1, 70) = 33.70, *p* < 0.001), and satisfaction (F(1, 70) = 26.50, *p* < 0.001) and more disengagement (F(1, 70) = 21.55, *p* < 0.001) compared with the non-clinical population
Le Grange et al. (2016) [80]	Australia	RCT	106 teenagers (average age = 15.5) →AN C = 93; A = 13 Family members (exact number not stated)	Chi-square tests Higher rates of remission of AN symptoms after implementation of Parent-Focused Therapy than after FBT (Wald chi-square = 5.85; df = 1; *p* = 0.016), but no statistically significant difference 6 months later (Wald chi-square = 3.75; df = 1; *p* = 0.053)
Lindstedt et al. (2020) [81]	Sweden	Naturalistic	1899 teenagers (average age = 16.1) C = 94.1%; A = 5.9% 55.3%→AN and 474 teenagers (average age = 16.3) C = 98.1%; A = 1.9% 61.6%→AN	Clustering One year after the end of the intervention, the majority of patients (77.4%), of all forms of treatment, reported better management of food, weight, and nutrition Over the same time period, FBT has the highest chance of disorder remission (49%), while individual therapy is most appropriate for adolescents.
Lock et al. (2017) [82]	USA	RCT	30 teenagers (average age = 14.49) →AN C = 24, A = 6	*t*-tests for independent samples Greater rates of eating disorder symptoms in participants in FBT combined with Art Therapy than with Cognitive Rehabilitation Therapy (t(28) = 2.26, *p* = 0.03) Exploratory data analysis Statistically significant correlation of changes in OCD features and changes in cognitive deficits (r = 0.59, *p* = 0.09) for both treatment combinations
Lock et al. (2016) [83]	USA	RCT	158 teenagers (average age = 15.3) →AN C = 89.2%; A = 10.8%	Intention-to-treat analysis Hospitalization rates after five weeks of intervention decrease more in FBT than in Systemic Therapy (U = 51.0, *p* = 0.02) Accounting regression Comorbid disorders predict early hospitalization (*p* = 0.03), whereas hospitalization negatively predicts weight improvement. (t = 52.6, *p* = 0.011). The findings apply to both forms of treatment
Matheson et al. (2022) [84]	USA and Canada	RCT	Total: 38 - FBT-V: 20 - GSH-FBT: 18	Improvement in eating-disorder symptoms: - GSH-FBT: All parents reported improvement - FBT-V: Improvement in mealtime behavior and mood/anxiety symptoms reported Worsening of symptoms: - GSH-FBT: More parents reported no worsening of symptoms - FBT-V: Some parents reported no worsening of symptoms Clinician comfort and competency: - GSH-FBT: Clinicians reported lower comfort and competency scores compared to FBT-V Logistical differences: - GSH-FBT required less clinician resources and positioned clinicians as coaches Clinician perspective: - Clinicians felt less comfortable and confident in delivering GSH-FBT compared to FBT-V, but their comfort with the coach role increased over time
McGowan et al. (2013) [85]	UK	RCT	Total: 126 - Intervention: 58 - Control: 68	- Increased automaticity in parental feeding behaviors: fruit-and-vegetable feeding habit increased by 1.0 points, healthy-snacks feeding habit by 1.8 points, and healthy-drinks feeding habit by 1.4 points in the intervention group compared to the control group. - Children’s food intake in the intervention group: increased fruit intake by 0.5 servings/day, vegetable intake by 0.8 servings/day, and healthy snack intake by 1.0 occasion/day; reduced unhealthy snack intake by 0.4 occasions/day; reduced sweet drink consumption by 0.6 occasions/day; and increased water intake by 0.6 occasions/day compared to the control group.
Milan and Acker, (2014) [86]	USA	Longitudinal	Total: 447	- Early attachment quality was not directly associated with disordered eating attitudes and behaviors (DEABs). - Among girls with an insecure attachment history: - Higher BMI at age 15 directly predicted more DEABs. - Maternal negative affect and pubertal weight gain indirectly predicted DEABs via greater preoccupation with parental relationships. - These direct and indirect paths did not occur among adolescent girls with a secure attachment history.
Murray et al. (2015) [87]	USA	Mixed method	40 teenagers (average age = 15.7) →BN C = 100%	*t*-tests for two dependent samples Reduction in symptoms of BN (t(68) = 4.52, *p* = 0.002), weight concerns (t(68) = 3.89, *p* = 0.001), and frequency of binge eating (t(68) = 2.19, *p* = 0.040), and an increase in emotion management skills (t(68) = 2.43, *p* = 0.045) at discharge relative to hospital admission
Pötzsch et al., (2018) [88]	Germany	Longitudinal	90 teenagers (average age = 14.58) C = 71; A = 19 40→BED 25→Overweight people—control team 25→Persons of normal weight—control group 90 mothers	Analysis of variance (ANOVA) Higher rates of perceived weight-related parental teasing in adolescents with BED than in overweight and normal weight (F(2, 89) = 8.37, *p* < 0.001, η2 = 0.16) Mediation Analysis Adoption of weight bias mediates between parental weight teasing and adolescent eating disorder-related psychopathological symptoms (b = 0.38, *p* < 0.001/b = 0.52, *p* < 0.001/b = 0.26, *p* < 0.001)
Puls et al. (2018) [89]	Germany	Longitudinal	64 teenagers (mean age = 14.17) →BED C = 52; A = 12	Nested models Negative correlation of the attachment relationship with the therapist and expectations from therapy (z = −0.06, *p* < 0.001). Negative correlation of the therapeutic alliance with the number of binge-eating episodes and with loss of control (z = −0.23, *p* < 0.05) and positive correlation with the attachment relationship with the therapist (z = 11.64, *p* < 0.001)
Ramalho et al. (2021) [90]	Brazil	Explanatory	8 teenagers (average age = 15.875) C = 100% 4→AN (average age = 15.75) 4→BN (average age = 16) 12 parents (8 mothers; 4 fathers), 5 grandmothers, and 1 sister	Interpretive phenomenological analysis Food is a means of controlling the parent–adolescent relationship and the perception of the parent’s presence or absence. Food is the source of conflict, both within the nuclear family and between three generations
Rienecke et al. (2016) [91]	USA	Non-RCT	215 teenagers (mean age = 15.26) C = 201; A = 14 121→AN (average age = 14.42) C = 110; A = 11 54→BN (average age = 15.94) C = 53, A = 1 40→Major Depressive Disorder—control group (average age = 15.44) C = 38; A = 2 322 parents 197→Parents of teenagers with AN (106 mothers; 91 fathers) 82→Parents of teenagers with BN (54 mothers; 28 fathers) 43→Parents of teenagers with Major Depressive Disorder (30 mothers; 15 fathers) Brothers 1.4 (M.O)→Adolescent siblings with AN 1.5 (M.O)→Teenage siblings with BN 1.7 (M.O)→Teenage siblings with Major Depressing Disorder	Multivariate analysis of variance Higher levels of fathers’ critical comments toward adolescents with BN (M = 2.0, SD = 0.5; *p* = 0.001) or Major Depressive Disorder (M = 1.6, SD = 0.4; *p* = 0.006) than with AN (M = 0.2, SD = 0.2). Accordingly, mothers express more critical comments in cases of BN (M = 1.7, SD = 0.3; *p* = 0.003) than in cases of AN (M = 0.6, SD = 0.2) Less emotional, overinvolvement, and warmth of mothers when siblings are present (M = 0.5, SD = 0.1/M = 1.7, SD = 0.1) than when they are absent (M = 0.9, SD = 0.1; *p* = 0.004/M = 2.3, SD = 0.1; *p* = 0.004)
Rousseau et al. (2020) [92]	Canada	Longitudinal	181 Adolescents (average age = 14.88) →AN F = 170; M = 11	Latent Class Analysis (LCA) Adolescents in families with a generalized conflictual relationship show a higher mean at increased risk of developing eating disorders than those in families with minimal clinical problems (χ^2^= 30.542, *p* < 0.000). Accordingly, in the second category, they present, compared to the first, a lower mean in ineffectiveness (χ^2^ = 21.184, *p* < 0.000), interpersonal problems (χ^2^= 42.793, *p* < 0.000), and excessive control (χ^2^ = 13.320, *p* < 0.000)
Sadeh-Sharvit et al. (2018) [93]	USA	Explanatory	158 teenagers (average age = 15.3) →AN C = 141; A = 17 Parents (exact number not mentioned)	Independent Samples *t*-Tests and Intention-to-Treat Analysis Statistically significant increase in self-efficacy of parents who participated in FBT from the beginning of treatment to the eighth session (mothers: b = 09, t = 4.27, *p* = 0.000/fathers: b = 09, t = 3.60, *p* = 0.000) No statistically significant difference between adolescents, mothers, or fathers in FBT or Systemic Therapy regarding flexibility (*p* > 0.05) Regression An increase in maternal self-efficacy by the eighth session mediates between treatment outcome and adolescent patient weight gain by the tenth session, both in FBT (B = 1.96, CI = 0.52, 3.41, *p* = 0.008), as well as in Systemic Treatment (B = 1.45, CI = 0.47, 2.43, *p* = 0.004)
Sepulveda et al. (2017) [94]	Spain	Case study	8 teenagers (mean age = 14 ± 1.41) →AN C = 100% 10 parents (8 mothers and 2 fathers)	Non-parametric Wilcoxon signed rank tests Following intensive exposure to family-based CBT, adolescents with AN showed a statistically significant increase in weight (M = 5.51 ± 3.44) and BMI (M = 0.91 ± 0.55) and a decrease in relative psychopathology eating disorders (M = −1.33 ± 1.02), restrictive behaviors (M = −1.97 ± 1.58), eating concerns (M = −2.07 ± 1.88), shape concerns (M = −2.44 ± 1.56), and weight (M = −2.27 ± 1.13) of the body, as well as its control (M = −16.16 ± 14.82)
Spettigue et al. (2018) [95]	Canada	Mixed method	32 teenagers (mean age = 15.48) →AN C = 90.6%; A = 9.4% →22 (average age = 15.47) received olanzapine as complementary therapy →10 (mean age = 15.31) not received olanzapine—group control	Mixed-effect regression analysis The experimental group gained more weight per week than the control group (β = 0.60; t = 2.35, df = 22.84, *p* = 0.028) Mixed models No statistically significant difference between the two groups in rate of change in self-reported eating-disorder symptoms (β = −0.10; t = −0.12, *p* = 0.904)
Stefini et al. (2017) [96]	Germany	RCT	81 teenagers (average age = 18.7) →BN C = 100%	Power analysis No statistically significant difference between CBT and Psychodynamic Therapy in terms of disorder remission rates (χ^2^ = 0.05, *p* = 0.81) Analysis of variance (ANOVA) CBT shows slightly better results in regard to reducing binge-eating (d = 0.23) and purging behaviors (d = 0.26), while Psychodynamic Therapy did in reducing eating concerns (d = −0.35)
Tafà et al. (2017) [97]	Italy	Longitudinal	150 teenagers (average age = 15.5) C = 100% 50→AN 50→BN 50→BED 290 parents	Multivariate analysis of variance (MANOVA) Statistically significant differences between the three groups, regarding the parents’ risk of psychopathology, both in mothers (F(3, 147) = 57.114; *p* < 0.05) and in fathers (F(3, 147) = 47.152; *p* < 0.05). Hierarchical regression The risk of parental psychopathology is a predictive factor of the adolescent’s perception of the way the family functions in all groups (PSA: mothers→β= 0.72, *p* < 0.01; fathers→β = 0.81, *p* < 0.05. PSB: mothers→β = 0.99, *p* < 0.01; fathers→β = 0.83, *p* < 0.01. DY: mothers→β = 1.01, *p* < 0.05)
Terache et al. (2022) [98]	Belgium	Longitudinal	Total: 150 - Females: 144	- BMI improved over the time of Multi-Family Therapy (MFT) and remained improved at both the 6- and 12-month follow-ups. - Eating disorders symptomatology (measured by EDI-II) improved over the time of MFT and remained improved at both the 6- and 12-month follow-up. - Perceived family functioning (measured by FAD) improved over the time of MFT and remained improved at both the 6- and 12-month follow-ups. - Improvement in dimensions of family functioning (roles, communication, and general family functioning) mediated the improvement of several dimensions of symptomatology (ineffectiveness, impulsivity, social insecurity, and interpersonal distrust).
Trainor et al. (2019) [99]	USA	RCT	95 teenagers (average age = 15.81) →AN C = 82; A = 13 Family members (exact number not stated)	Logistic regression Increased odds of having a comorbid disorder at the end of FBT (b = 2.00, *p* < 0.05) if multiple comorbid diagnoses were present at the start of the intervention Reduced odds of a comorbid disorder at the end of Family Therapy if psychotropic medication was used to treat Generalized Anxiety Disorder (b = −1.63, *p* = 0.04)
Van Doornik et al. (2021) [100]	Netherlands	Longitudinal	69 teenagers (average age = 15.55) →AN C = 67; A = 2 69 teenagers—non-clinical population as a control group (mean age = 15.48) C = 67; A = 2	Multivariate analysis of variance (MANOVA) Adolescents with AN show, compared to their peers who do not suffer from the disorder, less satisfaction in areas of life (F (1, 136) = 21.71, *p* < 0.001, η^2^ *p* = 0.14)
Van Langenberg et al. (2018) [101]	Australia	Case study	7 teenagers (average age = 15.14) →AN C = 100% 14 parents (13 mothers; 1 father) 12 siblings (C = 10; A = 2)	Coding and comparisons of interviews The inclusion of siblings in Family-Based Therapy helps to improve their understanding of the disorder Patients, parents, and siblings have different perceptions of the siblings’ role in treatment.
Wallis et al. (2017) [102]	Australia	RCT	57 teenagers (average age = 14.72) →AN C = 100% Parents (exact number not mentioned)	Regression Adolescents with AN who report lower levels of more general family functioning present, at the start of treatment, statistically significant comorbidity effects with depression (β = 1.92, *p* = 0.006), anxiety disorders (β = 2.40, *p* = 0.002), and symptoms of obsessive–compulsive disorder (β = 14.21, *p* = 0.001) Accounting regression Better levels of overall family functioning (β = −2.69, *p* = 0.005), communication (β = −1.69, *p* = 0.043), and problem-solving (β = −1.68, *p* = 0.029) are associated with an increased likelihood of remission disorder in the twentieth session, but not after a year
Walsh et al. (2018) [103]	USA	Explanatory	158 teenagers (average age = 15.3) →AN C = 141; A = 17 Teenage families (exact number of members not stated)	Linear regression models The maladaptive perfectionism of adolescent patients at the beginning of treatment shows a statistically significant relationship with the appearance of eating disorder symptoms at the end (β = 0.517, *p* < 0.001), as well as 6 (β = 0.371, *p* < 0.001) and 12 (β = 0.311, *p* = 0.001) months later
White et al. (2015) [104]	United Kingdom	RCT	21 teenagers (average age = 15.14) →AN C = 20; A = 1 36 parents (21 mothers; 15 fathers) 6 relatives—no siblings Participation of siblings (exact number not stated)	Bilateral analysis Statistically significant negative correlation between three parenting strategies for eating and adolescents’ positive general comments (r > 0.60) Statistically significant positive correlation between parental strategies for eating and the consumption of small amounts of food by adolescents (in four out of five strategies: r > 0.60—in one of the four, the correlation is acceptable only for mothers, and in another, only for fathers)
Wufong et al. (2019) [105]	Australia	Case study	13 parents of adolescents with AN (9 mothers; 4 fathers)	Discourse analysis Through the provision of guidelines for FBT, family functioning is improved, and parents’ anxiety about their child is temporarily reduced. However, this particular form of intervention contributes to increased parental guilt at the beginning of treatment and parental fears for adolescent well-being at the end of treatment.

AN = Anorexia Nervosa. BN = Bulimia Nervosa.

**Table 2:** AMSTAR-2 quality assessment of the studies (N = 46).

Reference	Risk of Bias	Inconsistency	Indirectness	Imprecision	Publication Bias	Quality of Evidence
Accurso et al. (2014) [60]	Low	Low	Low	Low	Low	High
Balottin et al. (2018) [61]	Moderate	Low	Low	Low	Low	Moderate
Baudinet et al. (2023) [62]	Low	Low	Low	Low	Low	High
Byrne et al. (2015) [63]	Moderate	Moderate	Low	Moderate	Low	Moderate
Ciao et al. (2015) [64]	Moderate	Moderate	Low	Moderate	Low	Moderate
Craig et al. (2019) [65]	Moderate	Moderate	Low	Moderate	Low	Moderate
Criscuolo et al. (2020) [66]	Low	Low	Low	Low	Low	High
Dalle Grave et al. (2015) [67]	Low	Low	Low	Low	Low	High
Dalle Grave et al. (2020) [68]	Low	Low	Low	Low	Low	High
Durfense et al. (2019) [69]	Low	Low	Low	Low	Low	High
Egbert et al. (2023) [70]	Moderate	Low	Low	Moderate	Low	Moderate
Fisher and Bushlow (2015) [71]	Moderate	Low	Low	Moderate	Low	Moderate
Godart et al. (2022) [72]	Low	Low	Low	Low	Low	High
Gorrell et al. (2018) [73]	Moderate	Moderate	Low	Moderate	Low	Moderate
Gorrell et al. (2020) [74]	Moderate	Moderate	Low	Moderate	Low	Moderate
Hilbert et al. (2019) [75]	Low	Low	Low	Low	Low	High
Hughes et al. (2017) [76]	Low	Low	Low	Low	Low	High
Hughes et al. (2014) [77]	Low	Low	Low	Low	Low	High
Hurst and Zimmer-Gembeck (2019) [78]	Low	Low	Low	Low	Low	High
Laghi et al. (2015) [79]	Moderate	Low	Low	Low	Low	Moderate
Le Grange et al. (2016) [80]	Low	Low	Low	Low	Low	High
Lindstedt et al. (2020) [81]	Low	Low	Low	Low	Low	High
Lock et al. (2017) [82]	Low	Low	Low	Low	Low	High
Lock et al. (2016) [83]	Low	Low	Low	Low	Low	High
Matheson et al. (2022) [84]	Moderate	Moderate	Low	Moderate	Low	Moderate
McGowan et al. (2013) [85]	Moderate	Moderate	Low	Moderate	Low	Moderate
Milan and Acker (2014) [86]	Moderate	Moderate	Low	Moderate	Low	Moderate
Murray et al. (2015) [87]	Moderate	Moderate	Low	Moderate	Low	Moderate
Pötzsch et al. (2018) [88]	Moderate	Moderate	Low	Moderate	Low	Moderate
Puls et al. (2018) [89]	Moderate	Moderate	Low	Moderate	Low	Moderate
Ramalho et al. (2021) [90]	Moderate	Moderate	Low	Moderate	Low	Moderate
Rienecke et al. (2016) [91]	Moderate	Moderate	Low	Moderate	Low	Moderate
Rousseau et al. (2020) [92]	Moderate	Moderate	Low	Moderate	Low	Moderate
Sadeh-Sharvit et al. (2018) [93]	Low	Low	Low	Low	Low	High
Sepulveda et al. (2017) [94]	Moderate	Moderate	Low	Moderate	Low	Moderate
Spettigue et al. (2018) [95]	Moderate	Moderate	Low	Moderate	Low	Moderate
Stefini et al. (2017) [96]	Low	Low	Low	Low	Low	High
Tafà et al. (2017) [97]	Moderate	Moderate	Low	Moderate	Low	Moderate
Terache et al. (2022) [98]	Moderate	Moderate	Low	Moderate	Low	Moderate
Trainor et al. (2019) [99]	Low	Low	Low	Low	Low	High
Van Doornik et al. (2021) [100]	Moderate	Moderate	Low	Moderate	Low	Moderate
Van Langenberg et al. (2018) [101]	Moderate	Moderate	Low	Moderate	Low	Moderate
Wallis et al. (2017) [102]	Low	Low	Low	Low	Low	High
Walsh et al. (2018) [103]	Low	Low	Low	Low	Low	High
White et al. (2015) [104]	Moderate	Moderate	Low	Moderate	Low	Moderate
Wufong et al. (2019) [105]	Moderate	Moderate	Low	Moderate	Low	Moderate

**Table 3:** Initial categorization of articles in systematic analysis (*n* = 46).

Parameters	Measurement Scale	*n*
Perceived Self-Efficacy	Generalized Self-Efficacy Scale	2 [63,76]
Self-Esteem	Rosenberg Self-Esteem Scale (RSE)	4 [61,69,79,86]
Weight–BMI–Height–Physical Appearance	Perceived parental weight teasing	1 [88]
	Weight Bias Internalization Scale (WBIS)	1 [88]
	Behaviors Toward Obese People Scale (ATOP)	1 [89]
	Obese Beliefs Scale	1 [89]
	Weight—treatment goal	1 [60]
	Pounds	3 [60,63,73]
	Calibrated digital scales	2 [60,63]
	Mass Balance Scale	1 [83]
	Ideal Body Weight	1 [60]
	50th percentile of Body Mass Index for age and sex according to the Centers for Disease Control and Prevention	8 [60,63,68,70,75,76,77,80]
	Wall stadiometer	1 [60]
	Calibrated wall stadiometer	2 [60,63]
	Body Checking Questionnaire	1 [70]
General Life Satisfaction	Anorexia Nervosa and Bulimia Nervosa Scoring Scale (SCANS)	1 [64]
Eating Disorders	Eating Disorders Investigation Questionnaire (EDE-Q)	23 [60,61,62,63,64,65,66,67,68,69,70,71,72,73,74,75,76,77,79,80,81,82,93,98]
	Eating Disorder Inventory (EDI) questionnaire	3 [60,63,79]
	Yale–Brown–Cornell Eating Disorder Scale (YBC-EDS)	4 [60,64,70,73]
	Eating Disorder Symptom Severity Scale (EDS)	1 [82]
	Structured Clinical Interviews for DSM-V Disorders	1 [77]
Skills	Wechsler Brief Intelligence Scale	1 [63]
	Rey–Osterrieth Complex Figure Test	1 [82]
	Wisconsin Card Sorting Job	1 [82]
Therapy	Treatment Appropriateness and Patient Expectations Scale (TSPE)	2 [77,84]
	Re-evaluation	1 [60]
	Follow-up by a therapist	1 [72]
	Patient Satisfaction Questionnaire	1 [81]
	Early patient withdrawal from treatment	2 [64,80]
	Visual Analogue Scales (VAS)	1 [84]
	Brief Multidimensional Students Life Satisfaction Scale—Peabody Treatment Progress Battery Version (BMSLSS-PTPB)	1 [70]
	Psychiatric and medical hospitalization number	1 [83]
	Length of hospital stay (weeks of treatment)	1 [83]
	Semi-structured interviews	2 [90,101]
Family	Parents Versus Anorexia Scale	3 [77,80,83]
	Assessment of Family and Bonding with Parents and Peers	1 [61]
	Family Adaptability and Cohesion Assessment Scale (FACES)	5 [61,66,79,97,102]
	Family Communication Scale	2 [79,97]
	Family Satisfaction Scale	2 [79,92]
	Standardized Clinical Family Interview (SCFI)	1 [61]
	Lausanne Trilogue Play	2 [61,66]
	Collaborative Parent and Family Rating System (CFRS)	1 [93]
	Self-Expressivity in the Family Questionnaire (SEFQ)	1 [66]
	Parent and Peer Attachment Inventory Scale (IPPA)	1 [86]
	Children’s Perceptions of Interparental Conflict Scale	1 [86]
	Pediatric Quality of Life Enjoyment and Satisfaction Questionnaire (PQ-LES-Q)	1 [90]
	Family Meal Coding System—Adolescent (FMCS-A)	1 [104]
	Helping Relationship Questionnaire (HRQ)	2 [87,94]
	Family Environment Scale	1 [61]
	Five Minute Speech Sample	1 [91]
	Clinical files	1 [60]
	Videos	1 [82]
	Images’ extraction	1 [90]
	Semi-structured interviews	2 [90,101]
Personality	Child and Adolescent Perfectionism Scale	1 [69]
	Borderline Personality Questionnaire (BPQ)	1 [69]
	Frost Multidimensional Perfections Scale (FMPS)	1 [69]
	Million Adolescent Clinical Inventory (MACI)	1 [66]
	Minnesota Multiphasic Personality Inventory	1 [71]
	Temperament and Character Inventory (TCI)	1 [69]
	Eysenck Personality Inventory (EPI)	1 [69]
Emotion	Positive and Negative Affect Scale—Expanded Scale (PANAS-X)	1 [65]
	Difficulties in Emotion Regulation Scale (DERS)	1 [63]
	Brief Symptom Inventory (BSI)	1 [60]
	Videotaped interviews	1 [61]
Comorbidity	Beck Depression Inventory (BDI)	7 [60,63,64,67,70,80,95]
	Kiddie Schedule for Affective Disorders and Schizophrenia (K-SADS)	5 [60,75,83,93,99]
	Revised Children’s Anxiety and Depression Scale (RCADS)	1 [83]
	Obsessional Compulsive Inventory—Revised (ChOCI-R)	1 [73]
	Symptoms Checklist (SCL)	1 [92]
	Symptoms Checklist 90 (SCL-90)	3 [60,68,91]
	Multidimensional Anxiety Scale for Children (MASC)	1 [80]
	State–Trait Anxiety Inventory (STAI)	1 [78]
	Beck Anxiety Inventory	1 [69]
	Mini International Neuropsychiatric Interview for Children and Adolescents (MINI-Kid)	3 [75,80,91]
	Clinical Global Impression—Severity (CGI-S)	1 [80]
	Quick Inventory of Depressive Symptomatology (QIDS)	1 [80]
Health	Obsessive–Compulsive Scale for Children (CY-BOCS)	4 [60,67,76,80]
	Evaluation of Clinical Problems	2 [60,80]
	Child Depression Inventory (CDI)	2 [60,80]
	Clinical Health Questionnaire (CHQ)	1 [88]
	Morgan–Russell Score Evaluation	1 [92]

## Data Availability

No new data were created or analyzed in this study. Data sharing is not applicable to this article.

## Abbreviation

ADHD	Attention Deficit Hyperactivity Disorder
AN	Anorexia Nervosa
BED	Binge-Eating Disorder
BMI	Body Mass Index
BN	Bulimia Nervosa
CBT	Cognitive–Behavioral Therapy
FBT	Family-Based Therapy
